# Enzymatic confinement in lipidic mesophases and liquid-crystalline nanoparticles: from nanostructure to biocatalytic function

**DOI:** 10.1007/s12551-026-01463-6

**Published:** 2026-10-03

**Authors:** Juliana Raw, Leticia M. Zanphorlin, Leandro R. S. Barbosa

**Affiliations:** 1 Institute of Physics, University of São Paulo, São Paulo, Brazil; 2 Brazilian Biorenewables National Laboratory (LNBR), Brazilian Center for Research in Energy and Materials (CNPEM), Campinas, SP Brazil

**Keywords:** Lipidic liquid-crystalline mesophases, Enzyme confinement, Nanoconfinement, Biocatalysis, Biorefineries, Nanotechnology, Lipidic nanoparticles, Nanoparticle characterization

## Abstract

Lipidic liquid-crystalline mesophases and their dispersed counterparts, lipid liquid-crystalline nanoparticles (LCNPs), constitute highly organized soft nanostructured materials that can accommodate biomolecules within both interconnected aqueous channels and lipid bilayers. Their internal architecture can be tuned over the nanometer scale, providing controlled microenvironments that regulate molecular transport, hydration, confinement, and interfacial interactions. These properties have established lipidic mesophases as versatile platforms for drug delivery and have also enabled new opportunities in biocatalysis, biosensing, membrane protein research, and functional nanobiotechnology. This review examines enzyme confinement in bulk lipidic mesophases and LCNPs from a biophysical perspective, relating mesophase structure to catalytic performance. We discuss how aqueous channel geometry, network topology, hydration, and lipid–protein interactions influence enzyme accommodation, diffusion, conformational stability, and activity. We further examine recent advances in enzyme-loaded lipidic systems, their emerging applications, and future opportunities in molecular bioeconomy and sustainable biorefineries. This body of evidence supports a conceptual framework in which enzyme behavior emerges from the combined influence of geometric constraints imposed by the aqueous network and physicochemical interactions at the enzyme–lipid interface.

## Introduction

Enzymes are highly selective and efficient biological catalysts that operate under mild conditions and are increasingly central to sustainable chemical manufacturing, pharmaceutical synthesis, food processing, biosensing, diagnostics, and biotechnology (Farhan et al. 2025). Their ability to accelerate reactions with high chemo-, regio-, and stereoselectivity makes them attractive alternatives to conventional catalysts, particularly in processes aligned with green chemistry, renewable carbon valorization, and low-energy production (Sheldon and Pelt 2013; Maghraby et al. 2023; Holyavka et al. 2023; Rade et al. 2023; Generoso et al. 2025; Miyamoto et al. 2022; Avila et al. 2026).

Despite these advantages, the practical use of free enzymes is often limited by insufficient operational stability, sensitivity to temperature, pH, solvents, interfaces, and proteolysis, as well as by difficulties in recovery, reuse, and integration into continuous processes (Maghraby et al. 2023). Enzyme immobilization has therefore become a key enabling strategy in applied biocatalysis, not only to facilitate catalyst recovery and reuse, but also to modulate the enzyme microenvironment, alter substrate accessibility, and improve compatibility with non-natural reaction conditions (Holyavka and Artyukhov 2025).

This perspective is particularly relevant for biomass-rich and biodiversity-rich economies, where the transition from a resource-based bioeconomy to a molecular bioeconomy depends on the ability to transform renewable biological resources into defined chemicals, materials, fuels, ingredients, and functional molecules (Wohlgemuth 2024). In this context, Brazil provides a representative example of such opportunity: its large agroindustrial base, lipid-rich feedstocks, lignocellulosic residues, microbial and plant biodiversity, and consolidated fermentation infrastructure create a unique landscape for enzyme-enabled biomanufacturing (Valli et al. 2018; Sulis et al. 2025).

In this context, enzymes are not merely process additives, but molecular tools that connect biodiversity, biomass, and renewable carbon to selective chemical transformations. They can mediate hydrolysis, oxidation, reduction, esterification, transesterification, decarboxylation, and multienzymatic cascade reactions under mild conditions, enabling higher-value uses of oils, carbohydrates, lignocellulosic streams, agroindustrial residues, and bio-based intermediates (Farhan et al. 2025). However, many enzymes relevant to biomass conversion, lipid transformation, oxidative biocatalysis, and cascade reactions still face biotechnological limitations in macromolecule stability, substrate accessibility, interfacial inactivation, and operation in complex or multiphasic media (Porta et al. 2026). These challenges motivate the development of and increase interest in confinement platforms that may preserve enzyme structure while controlling hydration, substrate partitioning, and local molecular organization (Mendozza et al. 2020; Mezzenga 2023).

Most enzyme immobilization strategies rely on solid or semi-rigid materials, including inorganic oxides, mesoporous silica, polymers, hydrogels, magnetic nanoparticles, carbon-based materials, and metal–organic frameworks (Holyavka et al. 2023; Ma et al. 2025). These platforms can provide high surface area, mechanical robustness, chemical functionalization, and, in some cases, precise pore dimensions (Holyavka and Artyukhov 2025). However, enzyme performance after immobilization depends not only on loading capacity and support stability but also on local hydration, molecular crowding, substrate partitioning, product diffusion, interfacial interactions, and the preservation of the enzyme native-like conformational ensemble. Immobilization should therefore be viewed less as a passive anchoring strategy and more as a means to engineer catalytic microenvironments.

Lipidic mesophases offer a particularly attractive but still underexplored class of soft, biomimetic matrices for enzymatic confinement (Manni et al. 2020). Unlike inorganic supports, polymeric matrices, or other solid-phase platforms, lipid mesophases are formed by the self-assembly of amphiphilic molecules into liquid-crystalline architectures with nanometrically defined aqueous and hydrophobic domains (Angelova et al. 2011). The structural description of lipidic cubic phases has a long-standing foundation in lipid physical chemistry. Seminal studies by Luzzati and co-workers established that lipid–water systems can organize into three-dimensional periodic cubic architectures and demonstrated how their symmetry and structural dimensions can be resolved from X-ray diffraction patterns. In particular, the analysis by Mariani, Luzzati, and Delacroix provided a systematic structural description of cubic phases in lipid-containing systems and emphasized their potential biological relevance (Mariani et al. 1988). This early structural framework remains fundamental to the modern interpretation of inverse bicontinuous cubic phases by small-angle X-ray scattering.

In inverse bicontinuous cubic phases $Q_{II}$, typically described by the Pn$\bar{3}$m, Im$\bar{3}$m, and Ia$\bar{3}$d space groups, a continuous lipid bilayer separates two non-intersecting aqueous channel networks (Qiu and Caffrey 2000). In the inverse hexagonal phase ($H_{II}$), water channels are arranged in a two-dimensional hexagonal lattice and surrounded by a continuous lipid matrix. The sponge phase ($L_3$), in turn, is a disordered bicontinuous phase with higher flexibility and, in many cases, larger aqueous domains (Valldeperas et al. 2016). These mesophases can be described by parameters such as the lattice parameter, aqueous channel diameter, interfacial curvature, and water volume fraction, which are accessible by small-angle X-ray scattering and can be tuned by lipid composition, hydration, temperature, additives, and stabilizers (Valldeperas et al. 2019a; Mezzenga et al. 2019).

The relevance of these systems for enzymes arises from the fact that many catalytic processes are intrinsically sensitive to confinement, hydration, interfaces, and substrate partitioning (Mezzenga 2023). The internal aqueous channels of lipidic mesophases can accommodate soluble enzymes, substrates, cofactors, and products, while the hydrophobic domains can partition apolar substrates or provide membrane-like environments for amphiphilic and membrane-associated enzymes (Valldeperas et al. 2019a). Thus, lipidic mesophases can influence catalysis through coupled effects on diffusion, water activity, substrate enrichment, interfacial stabilization, and conformational dynamics. Importantly, these effects depend not only on lipid composition but also on the topology and size of the internal aqueous network. Enzymatic kinetics in liquid-crystalline mesophases has been shown to depend on both channel size and mesophase topology (Sun et al. 2015), while lipidic cubic phases can preserve the activity and enhance the stability of membrane-bound enzymes by providing a biomimetic bilayer-rich environment (Sun et al. 2016).

Lipidic mesophases have already had a major impact in neighboring fields. In structural biology, lipidic cubic phases are widely used for in meso crystallization of membrane proteins, where the mesophase serves as both a membrane mimic and a crystallization medium (Caffrey and Cherezov 2009; Caffrey 2015). In drug delivery, dispersed lipid liquid-crystalline nanoparticles, including cubosomes and hexosomes, have been extensively investigated as carriers for hydrophilic, hydrophobic, and amphiphilic therapeutic molecules because of their high internal surface area, tunable nanostructure, and biocompatibility (Barriga et al. 2019; Leu et al. 2023; Palma et al. 2023). In bioelectrochemistry, enzymes confined in lipidic cubic phases on electrode surfaces have been explored since the pioneering work of Razumas and co-workers, demonstrating their potential in amperometric biosensors and direct electron-transfer systems (Razumas et al. 1994; Grippo et al. 2019).

When dispersed into colloidal particles, these mesophases form lipid liquid-crystalline nanoparticles, including cubosomes, hexosomes, and sponge-phase nanoparticles. These particles retain key features of the parent bulk mesophase while offering colloidal dimensions, large internal interfacial area, aqueous nanochannels, and compatibility with biological media (Angelova et al. 2011).

Cubosomes are commonly stabilized using Poloxamer 407 (Pluronic^®^ F127, BASF), a triblock copolymer composed of poly(ethylene oxide)-poly(propylene oxide)-poly(ethylene oxide) (PEO-PPO-PEO). Stabilizers such as Poloxamer 407 are used to preserve colloidal stability and internal order, producing particles often in the 100–400 nm range, although size and internal structure depend strongly on composition and preparation method (Barriga et al. 2019). For enzymatic applications, this combination is conceptually powerful: the enzyme is not merely carried by the nanoparticle, but confined within a soft nanostructured environment whose geometry, hydration, and interfacial properties can directly influence catalytic behavior.

Nevertheless, the use of lipid liquid-crystalline nanoparticles as enzyme-confining platforms remains much less developed than their use in drug delivery or membrane-protein crystallization. Several fundamental questions remain unanswered:

- How does mesophase topology affect enzyme loading, retention, activity, and apparent kinetics?
- Which enzymes are favored by bicontinuous cubic, inverse hexagonal, or sponge-like environments?
- How do aqueous channel diameter, lipid curvature, and substrate partitioning influence turnover and stability?
- To what extent can structural parameters (like water channel diameter, unit cell parameter, among others) be correlated with catalytic parameters such as apparent $K_M$, $V_\textrm{max}$, turnover number, operational stability, and reusability?
- Finally, how can genuine nanoconfinement effects be distinguished from artifacts associated with enzyme leaching, partial unfolding, diffusion limitations, or changes in substrate availability?

### This review addresses these questions by discussing lipidic mesophases and lipid liquid-crystalline nanoparticles as functional platforms for enzymatic confinement.

Moreover, we examine recent advances in enzyme-loaded lipidic systems, their emerging applications, and future opportunities in molecular bioeconomy and sustainable biorefineries. Firstly, we describe the structural diversity of mesophases relevant to enzyme encapsulation, with emphasis on bicontinuous cubic, inverse hexagonal, and sponge phases. We then examine how internal geometry, aqueous channel dimensions, interfacial curvature, and lipid composition influence enzyme structure, stability, substrate accessibility, and catalytic performance. Finally, we propose an integrated biophysical framework in which SAXS, spectroscopies, microscopy, and activity assays are combined to establish structure–function relationships in enzyme–lipid mesophase systems.

**Fig. 1:**
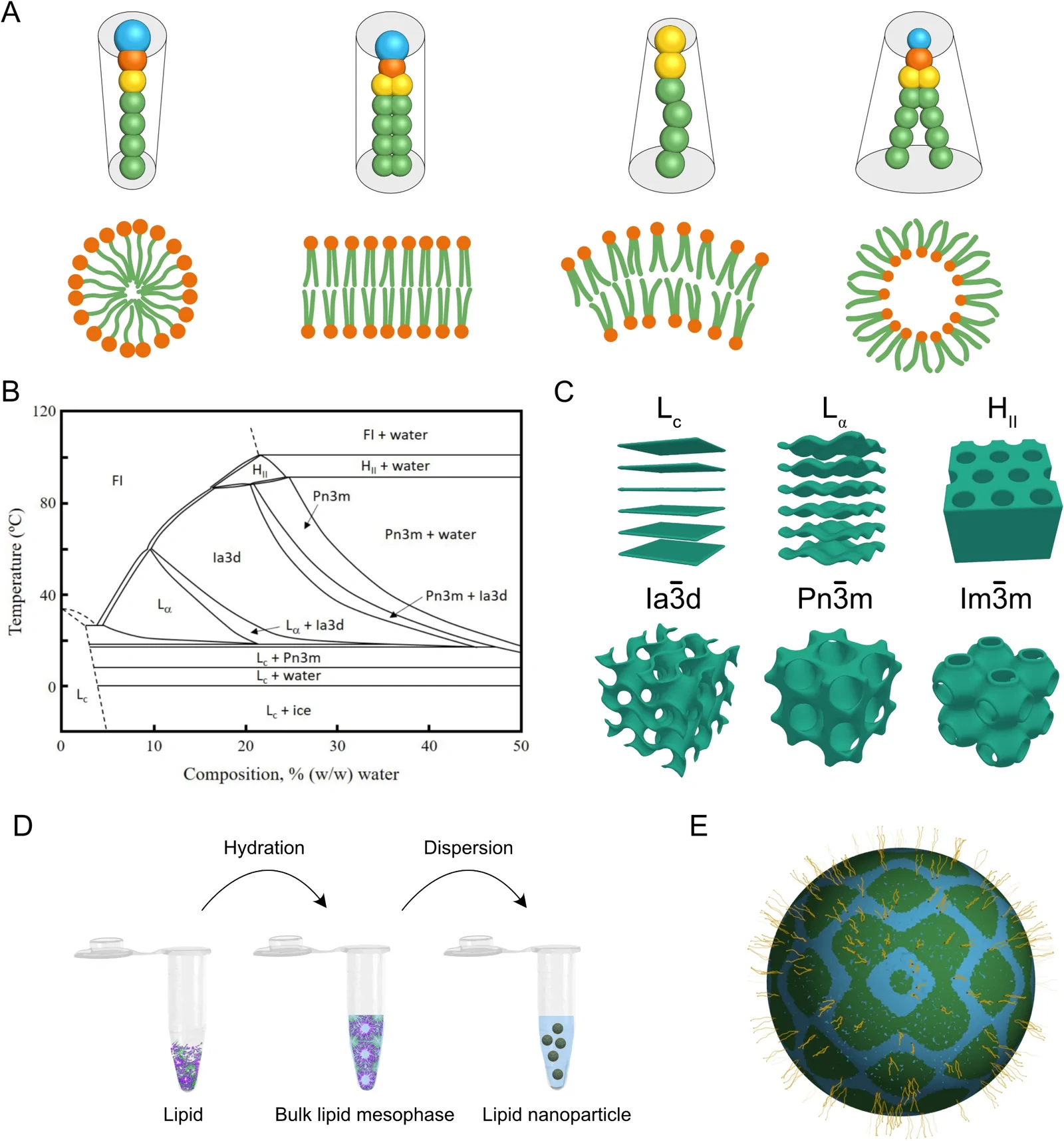
Structural landscape of lipidic mesophases and LCNPs. **A** Molecular geometry and the critical packing parameter (CPP) determining mesophase identity. **B** Temperature–composition phase diagram of the monoolein/water system, showing regions of $L_c$, $L_\alpha$, Ia$\bar{3}$d, Pn$\bar{3}$m, and $H_{II}$ stability. Adapted with permission (Qiu and Caffrey 2000). **C** Three-dimensional representations of $L_c$, $L_\alpha$, $H_{II}$, Ia$\bar{3}$d, Pn$\bar{3}$m, and Im$\bar{3}$m architectures. **D** Workflow from lipid hydration to bulk mesophase, colloidal dispersion, and lipid nanoparticle. **E** Schematic cubosome with internal Pn$\bar{3}$m cubic structure and Poloxamer 407 (F127) corona

To provide a structural reference before discussing enzyme confinement and its functional consequences, Fig. 1 summarizes the molecular organization, phase behavior, three-dimensional architectures, and formation of the principal lipidic mesophases and lipid liquid-crystalline nanoparticles discussed throughout this review.

## Lipidic mesophases and liquid-crystalline nanoparticles as enzymatic microenvironments

### Internal organization of lipidic mesophases

The self-assembly of amphiphiles in aqueous systems results from the delicate balance between opposing forces acting on the amphiphilic molecule. The hydrophobic effect favors chain packing to minimize contact with water, whereas steric and electrostatic repulsions between polar headgroups tend to expand the interfacial area per molecule (Israelachvili et al. 1977), since they may be able to form hydrogen bonds with water. The balance between these forces defines an optimal headgroup area, $a_0$. This area, combined with the volume of the hydrophobic chains, $v_\textrm{chain}$, and their critical length, $l_c$, determines the critical packing parameter, as Sarkar et al. (2019):

1 $$\begin{aligned} \textrm{CPP} = \frac{v_\textrm{chain}}{a_0 \times l_\textrm{chain}} \end{aligned}$$

$\textrm{CPP} \le 1/3$ favors spherical micelles, while values between 1/3 and 1/2 correspond to cylindrical aggregates, and values close to 1 correspond to lamellar bilayers (Nagarajan 2002; Eczacioglu et al. 2026). When $\textrm{CPP} > 1$, the spontaneous curvature becomes negative, stabilizing inverse mesophases, including inverse bicontinuous cubic, inverse hexagonal, and inverse micellar phases (Eczacioglu et al. 2026; Sarkar et al. 2019). Lipids with unsaturated chains and relatively small headgroups, such as monoolein and phytantriol, exhibit CPP values greater than 1 and spontaneously self-assemble into inverse liquid-crystalline mesophases in excess water, generating nanostructured aqueous microenvironments of controllable dimensions. Representative mesophase architectures are shown in Fig. 1A–C.

Among the mesophases formed by lipids with $\textrm{CPP} > 1$, inverse bicontinuous cubic phases are distinguished by the three-dimensional organization of a continuous lipid bilayer around two interpenetrating and non-interconnecting aqueous networks (Mariani et al. 1988; Mezzenga et al. 2019). Their structural description in terms of periodic minimal-surface-related architectures provides the basis for relating crystallographic symmetry, lattice dimensions, interfacial curvature, and aqueous-network geometry to the physicochemical properties of the mesophase. Three space groups are most commonly encountered: Pn$\bar{3}$m, Im$\bar{3}$m, and Ia$\bar{3}$d. Each is identifiable by X-ray diffraction experiments through characteristic ratios between Bragg peak positions (Caselli et al. 2024). From the unit cell parameter, *a*, which is easily obtained by SAXS, the aqueous channel diameter, $D_W$, can be calculated through geometric relationships that depend on the symmetry of the phase (Caselli et al. 2024).

The inverse hexagonal phase is formed by aqueous cylinders of circular cross-section surrounded by inverse lipid monolayers and arranged in a hexagonal lattice. The aqueous channels are parallel and non-interconnecting, restricting diffusion along the axis of each cylinder. The sponge phase, $L_3$, is a structurally disordered bicontinuous mesophase related to cubic phases but lacking long-range periodicity. Its SAXS profile exhibits diffuse peaks instead of crystalline Bragg peaks (Valldeperas et al. 2019b). The absence of periodic geometric restrictions gives the $L_3$ phase larger and more flexible aqueous channels, reaching diameters up to approximately 13 nm, making it more suitable for the confinement of high-molecular-weight enzymes than typical $Q_{II}$ or $H_{II}$ phases (Valldeperas et al. 2019a).

The aqueous channel diameter is a tunable parameter. In monoolein mesophases, in the absence of external additives, $D_W$ is typically between 3 and 4 nm. Incorporation of co-surfactants with less negative spontaneous curvature, such as octyl glucoside, modifies the effective CPP of the bilayer and promotes swelling of the aqueous network, expanding $D_W$ up to approximately 6–7 nm in Pn$\bar{3}$m phases of monoolein/octyl glucoside (Angelova et al. 2011). Electrostatic swelling using anionic phospholipids such as DSPG can further expand water channels to ${\sim }20$ nm in cubic phases of monopalmitolein (Zabara et al. 2018). The addition of cholesterol and zwitterionic phospholipids to monoolein systems, on the other hand, can generate highly swollen mesophases with exceptionally large lattice parameters. In the monoolein–cholesterol–DMPC system, the lattice parameter can reach ${\sim }35$ nm (Sarkar et al. 2019), corresponding to aqueous domains larger than the hydrodynamic diameter of many enzymes of biotechnological interest.

In addition to lipid composition, hydration degree and temperature are central physico-chemical parameters that determine the mesophase identity (Qiu and Caffrey 2000). These adjustable structural properties represent a significant advantage of lipid mesophases over fixed-geometry supports for enzymatic confinement. The relationship between molecular geometry, mesophase organization, and LCNP formation is summarized in Fig. 1.

### From bulk mesophases to lipid liquid-crystalline nanoparticles

The lipid mesophases described above exist primarily as bulk phases in excess water, but they can be dispersed into colloidal suspensions of nanoparticles in the presence of steric stabilizers. Two main approaches are commonly used: mechanical fragmentation of the bulk phase by high-pressure homogenization or sonication, known as the *top-down route*, and direct self-assembly from lipid–hydrotrope mixtures added to the aqueous phase, known as *the bottom-up route* (Palma et al. 2023). More recently, some researchers have also explored the possibility of producing cubosomes using microfluidic devices (Pilkington et al. 2023; Kim et al. 2018). A simplified scheme of bulk mesophase formation and nanoparticle dispersion is presented in Fig. 1D, E.

As mentioned earlier in the text, Poloxamer 407 is the most widely used stabilizer, where its slightly hydrophobic PPO segment anchors onto the surface of the lipid nanoparticle, whereas the hydrophilic PEO chains project into the external aqueous phase, providing steric repulsion and colloidal stability without penetrating the internal mesophase (Angelova et al. 2011).

**Fig. 2:**
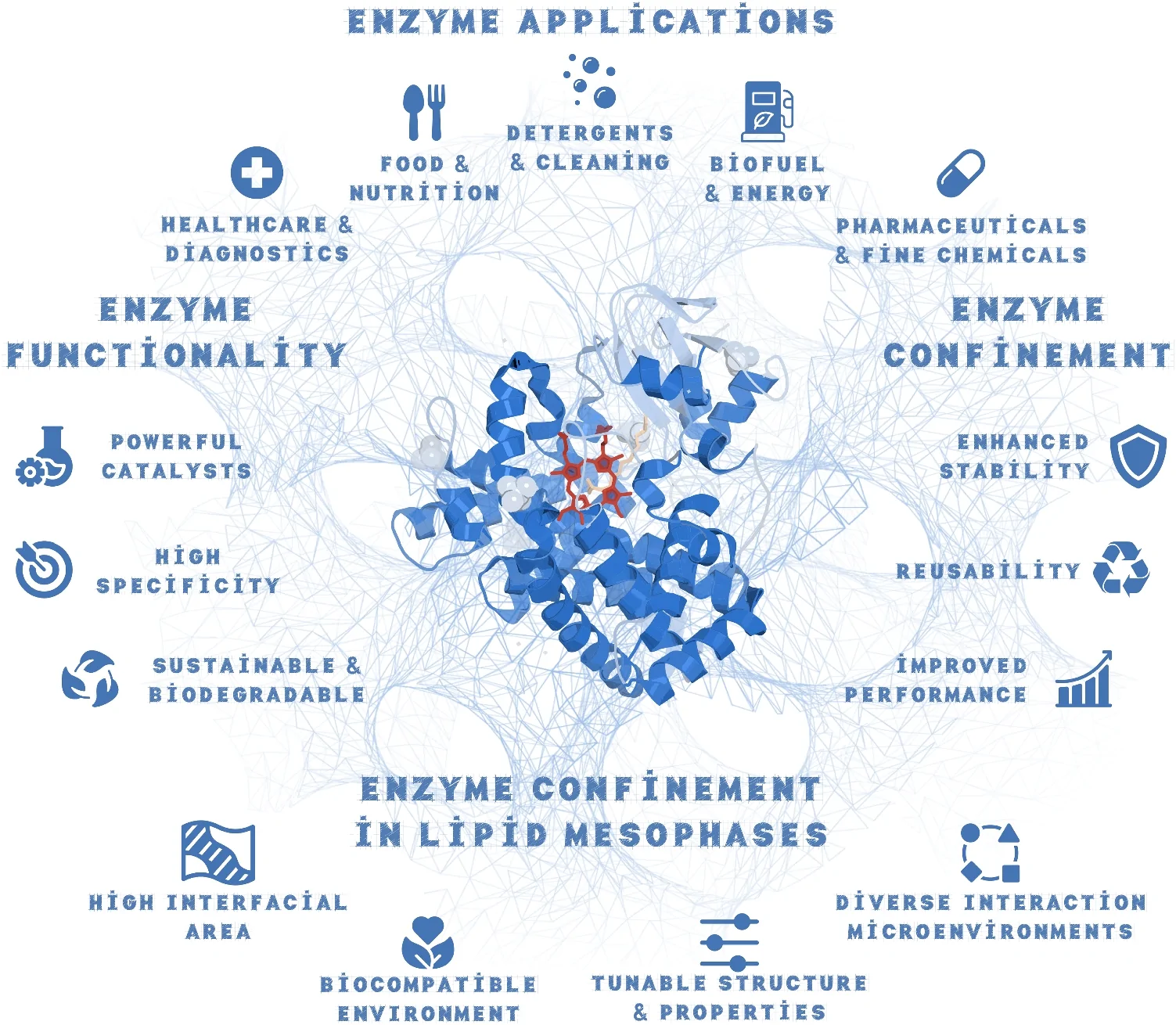
Overview of the central concepts addressed in this review, linking enzyme functionality and applications to the physicochemical properties of enzymatic confinement in lipidic mesophases

Enzymatic lipid hydrolysis can also enable the formation of ordered LCNPs through enzyme-driven reorganization of lipid assemblies. Lipase-triggered digestion converts initially disordered emulsions composed of digestible triglycerides and non-digestible lyotropic lipids into cubosomes or hexosomes, a process monitored in real time by synchrotron SAXS (Fong et al. 2014). The resulting internal nanostructure is governed by the ionization state of the fatty acids generated during lipolysis, with the cubic Pn$\bar{3}$m phase favored at intestinal pH and the inverse hexagonal phase stabilized under more acidic conditions (Hong et al. 2015). More recently, pancreatic lipase was shown to induce the transition from an oriented inverse hexagonal phase to an oriented bicontinuous cubic Pn$\bar{3}$m phase in supported GMO/triolein films, accompanied by progressive lattice swelling monitored by synchrotron GISAXS (Freire et al. 2024).

Following these procedures, dynamic light scattering (DLS) measurements typically yield hydrodynamic diameters that fall between 100 and 400 nm, with polydispersity indices below 0.2 in optimized formulations (Pilkington et al. 2023). The Poloxamer 407 concentration and dispersion conditions influence size and nanoparticle polydispersity, but the internal geometry is primarily determined by lipid composition, hydration degree, and temperature (Palma et al. 2023).

The characteristic Bragg peaks of the internal mesophase are generally maintained in dispersed LCNPs, preserving the lattice parameter and aqueous channel diameter of the parent bulk system (Angelova et al. 2011). This structural preservation is also observed in highly swollen systems (Sarkar et al. 2019). The finite size of the nanoparticles and the presence of the F127 layer broaden the Bragg peaks relative to bulk phases. However, functionalization steps such as enzyme loading can modify structural parameters or induce phase transitions. For this reason, structural characterization of LCNPs should be performed before and after enzyme incorporation (Valldeperas et al. 2019a; Liu et al. 2013).

Throughout this review, we distinguish explicitly between *bulk lipidic mesophases* and *dispersed LCNPs*. The former correspond to macroscopically continuous liquid-crystalline lipid–water matrices, whereas the latter are colloidal particles that retain an internal liquid-crystalline nanostructure while being dispersed in an external aqueous phase and stabilized by an interfacial polymer or surfactant layer. Although both systems share related internal geometries, they are not functionally equivalent. Finite particle dimensions, the presence of an external surface and stabilizer corona, colloidal stability, enzyme exchange with the surrounding medium, leakage, and external surface adsorption introduce additional processes in dispersed LCNPs that are absent or substantially different in bulk mesophases. Accordingly, throughout the following sections, we explicitly identify whether the experimental evidence discussed was obtained in a bulk mesophase or in a dispersed nanoparticle system.

Having established the structural relationship between bulk mesophases and dispersed LCNPs, the broader conceptual framework connecting enzyme functionality, nanoconfinement, and prospective applications is summarized in Fig. 2.

**Fig. 3:**
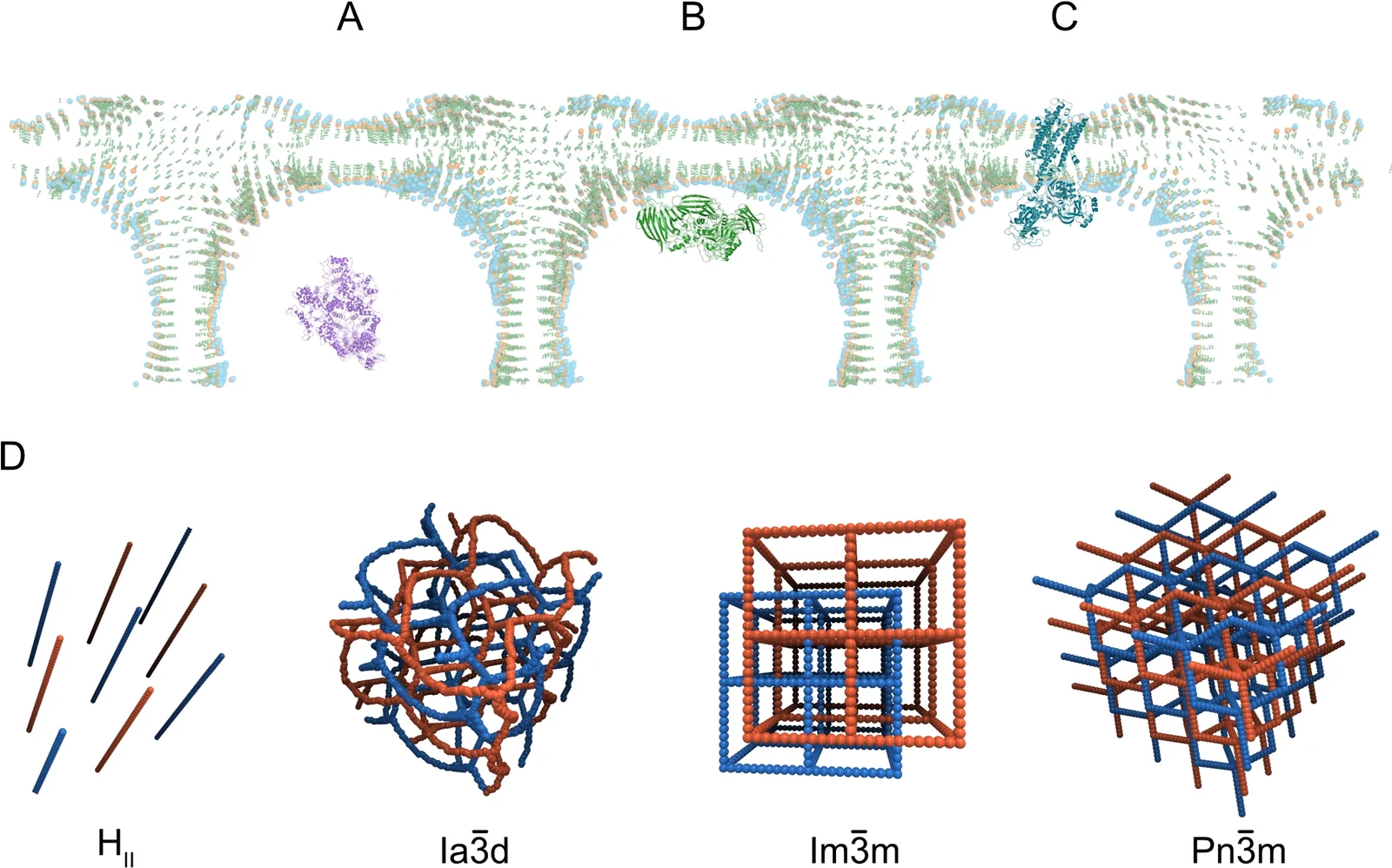
Enzyme localization within lipid liquid-crystalline nanoparticles. **A–C** Cross-sectional representations of enzyme positions: within aqueous channels (**A**), at the lipid–water interface (**B**), and inserted into the lipid bilayer, (**C**). **D** Aqueous channel network topologies in $H_{II}$, Ia$\bar{3}$d, Im$\bar{3}$m, and Pn$\bar{3}$m mesophases, illustrating differences in connectivity and dimensionality

### Enzyme accommodation and functional localization in lipidic mesophases and LCNPs

The accommodation of enzymes in bulk lipidic mesophases and dispersed lipid liquid-crystalline nanoparticles is a central determinant of their functional performance. Importantly, enzyme loading should not be interpreted as defining a single or mutually exclusive localization state. Rather, within a given LCNP population, enzyme molecules may be distributed among several coexisting microenvironments, including the aqueous channels, local defects within the internal network, the lipid–water interface, the lipid bilayer, and the external particle surface or stabilizer corona. The relative populations occupying these environments are expected to depend on enzyme size, surface charge, hydrophobicity, conformational flexibility, lipid composition, aqueous channel dimensions, and interfacial properties. Consequently, a measured encapsulation or association efficiency represents the total fraction of enzyme associated with the nanoparticle system, but does not by itself establish a unique localization within the internal nanostructure. These different localization modes, which may coexist within the same formulation, have distinct consequences for substrate accessibility, protection against degradation, enzyme leakage, conformational stability, and apparent catalytic activity. The principal localization modes discussed in this section are illustrated in Fig. 3A–C.

An important geometric criterion for evaluating compatibility between an enzyme and an LCNP is the relationship between the hydrodynamic diameter of the enzyme, $d_h$, determined by DLS, crystallography, or structural modelling, or the radius of gyration $R_g$ from solution SAXS (Spinozzi et al. 2014), and the aqueous channel diameter, $D_W$, of the mesophase. When $d_h < D_W$, the enzyme can, in principle, be accommodated entirely within the aqueous channels of the network. This configuration is particularly favorable for soluble enzymes that require a hydrated environment and for applications in which the lipid matrix is expected to protect the protein from denaturation, proteolysis, or external stress.

However, $d_h > D_W$ does not necessarily prevent enzyme incorporation (van’t Hag et al. 2017). In bicontinuous cubic phases, proteins larger than the average aqueous channel diameter can be confined within local network defects. These disordered regions at bilayer junctions act as nanopockets with dimensions larger than the average periodic channel and can host proteins that would otherwise be excluded by simple geometric criteria (Angelova et al. 2011). Such defect-mediated accommodation may generate heterogeneous enzyme populations, in which different fractions experience distinct hydration, diffusion, and interfacial environments.

In sponge-phase nanoparticles, encapsulation can also occur when the aqueous channel is smaller than the enzyme. Gilbert and co-workers showed that β-galactosidase, with $d_h = 11.8$ nm, was encapsulated with 100% efficiency in $L_3$ nanoparticles with $D_W = 8.6$ nm. This behavior is attributed to partial penetration of the enzyme into the lipid bilayer, favored by hydrophobic regions exposed on the protein surface (Gilbert et al. 2019).

In contrast, aspartic protease, with $d_h$ smaller than the corresponding $D_W$ of the $L_3$ phase, exhibited an encapsulation efficiency of 81%. Importantly, Raman spectroscopic changes involving aromatic amino-acid residues supported partial interaction of the enzyme with the lipid bilayer rather than a purely aqueous-channel localization (Valldeperas et al. 2019b). This example illustrates why encapsulation efficiency alone cannot define the microscopic localization of the enzyme. These examples show that protein–lipid affinity can override purely geometric expectations and that interfacial localization may be particularly relevant for enzymes acting on bulky, hydrophobic, or amphiphilic substrates.

Enzyme loading can also induce phase transitions, demonstrating that enzymes are active components capable of perturbing the geometric equilibrium of the mesophase. In GMO/Poloxamer 407 cubosomes loaded with earthworm fibrinolytic enzyme, a Pn$\bar{3}$m-to-Im$\bar{3}$m transition was accompanied by changes in the lattice parameter, attributed to competition between the protein and lipid headgroups for interfacial hydration (Liu et al. 2013).

Similarly, catalase-loaded cubosomes exhibited a Pn$\bar{3}$m-to-Im$\bar{3}$m transition with an expansion of the lattice parameter by 3 to 6 nm relative to the blank formulation, interpreted as protein-induced osmotic swelling of the cubic lattice (Rakotoarisoa et al. 2019).

In sponge-phase systems, aspartic protease induced an $L_3$-to-Pn$\bar{3}$m transition at high concentrations, whereas β-galactosidase, despite its larger volume, did not perturb the $L_3$ phase (Valldeperas et al. 2019b; Gilbert et al. 2019). Together, these observations indicate that the structural response of the mesophase is specific to each enzyme–lipid pair and cannot be predicted solely from protein size or concentration (Mazzoni et al. 2016).

Quantifying enzyme association or encapsulation efficiency through indirect methods, such as differential centrifugation and ultrafiltration, requires careful interpretation. These approaches can distinguish particle-associated protein from protein remaining free in the surrounding aqueous phase, but they do not establish where the associated enzyme resides within the lipidic system. In particular, a high encapsulation efficiency should not be considered, by itself, evidence of true nanoconfinement.

The measured associated fraction may include enzymes located within aqueous channels or internal defects, enzymes partially inserted at the lipid–water interface or within the bilayer, and, in dispersed LCNPs, enzymes adsorbed onto the external particle surface or stabilizer corona. Therefore, the terms *association*, *encapsulation*, and *nanoconfinement* should not be regarded as experimentally equivalent unless additional localization evidence is available. Establishing enzyme localization therefore requires converging evidence from complementary structural, spectroscopic, colloidal, and functional measurements. SAXS can reveal enzyme-induced changes in lattice parameter, aqueous-channel dimensions, or mesophase symmetry and thereby demonstrate coupling between protein incorporation and the internal nanostructure, although such changes do not uniquely determine the enzyme position. Changes in zeta potential can provide indirect evidence of enzyme association with the external particle interface, particularly when substantial surface-charge shifts occur after loading. Vibrational spectroscopy, including Raman and infrared spectroscopy, can probe changes in the local environment of amino-acid residues and support interfacial or bilayer-associated localization. Fluorescence quenching by probes that preferentially partition into the lipid bilayer can provide more direct evidence that specific protein domains are located near or within the hydrophobic region. In addition, protease-protection experiments, leakage or release assays, and inhibitor- or substrate-accessibility measurements can provide functional evidence that distinguishes protected internalized populations from externally accessible enzyme fractions. No single technique, however, generally provides an unambiguous localization map, and the strongest assignments arise from combinations of independent measurements.

This distinction has direct consequences for the interpretation of functional data. Only the enzyme fraction effectively internalized or integrated at the interface undergoes true confinement within the mesophase, protection against proteolysis, changes in hydration, or diffusion restrictions imposed by the aqueous network. In contrast, externally adsorbed enzymes may contribute to apparent activity without reflecting the catalytic behavior of the confined population. Therefore, for enzyme-based applications, a complete picture requires information on how much enzyme is associated with the LCNP, which fraction is catalytically active, how stable it remains, how the mesophase architecture controls substrate access and product release, and where the enzyme resides within the particle. However, directly determining the location within dispersed LCNPs remains a methodological challenge.

**Table 1:** Representative structure–function relationships between lipidic mesophase architecture and enzymatic behavior. The table emphasizes the structural variables that have been experimentally correlated with kinetic parameters, lag time, catalytic efficiency, or operational stability. $D_W$: aqueous channel diameter; $d_h$: hydrodynamic diameter; $K_M$: Michaelis constant; $V_{\textrm{max}}$: maximum reaction velocity; η: effectiveness factor

System	Mesophase	Enzyme	Structural variable	Impact on kinetics or functional stability	Ref.
Bulk phytantriol	Pn$\bar{3}$m, Im$\bar{3}$m	Alkaline phosphatase	$D_W=2.3$–3.6 nm	For substrate confinement, the conversion rate was approximately 31-fold lower than in free solution at $D_W=2.3$ nm and 14-fold lower at 3.6 nm. When the enzyme was confined, a 5–10 min lag period was observed and decreased with increasing $D_W$	Mendozza et al. (2020)
Bulk monoglyceride	Pn$\bar{3}$m	Horseradish peroxidase	$D_W=3.5 \rightarrow 7.2$ nm	Increasing $D_W$ reduced lag time from 1260 to 119 s and decreased apparent $K_M$ from 7.5 to 3.0 mM. The apparent $k_{\textrm{cat}}$ increased from 35.2% to 92.1% of the value measured in solution	Sun et al. (2014)
Bulk monolinolein	Pn$\bar{3}$m, Ia$\bar{3}$d, $H_{II}$	Horseradish peroxidase	Network topology at constant $D_W \approx 3.4$ nm	At equivalent channel diameter, relative activity followed Pn$\bar{3}$m (100%) > Ia$\bar{3}$d (38.5%) > $H_{II}$ (15.1%). Lag times were approximately 0, 1082, and 1960 s, respectively, demonstrating an independent effect of network connectivity	Sun et al. (2015)
Bulk $L_3$ and dispersed $L_3$-NPs	Sponge $L_3$	β-Galactosidase	$D_W=8.6$ nm; $d_h=11.8$ nm	Apparent $K_M$ increased from 1.9 to 2.9 mM and $V_{\textrm{max}}$ decreased from 0.193 to 0.104 ΔA min$^{-1}$, giving $\eta \approx 0.5$. The confined enzyme also showed slower deactivation than the free enzyme	Gilbert et al. (2019)
Bulk monolinolein	Pn$\bar{3}$m	D-Fructose dehydrogenase	$D_W\approx 3.2$ nm; membrane–bilayer compatibility	$K_M$ remained close to the solution value (1.7 vs. 1.3 mM). The enzyme retained 100% activity after 5 days and 63% after 10 reuse cycles, contrasting with the stronger functional restriction observed for soluble HRP in the same type of mesophase	Sun et al. (2016)
Bulk $L_3$ and dispersed $L_3$-NPs	Sponge $L_3$	Aspartic protease	$D_W\approx 8$ nm; enzyme–bilayer interaction	The enzyme remained catalytically active after confinement and showed improved temporal stability relative to the free enzyme. At high protein concentration, enzyme incorporation induced an $L_3\rightarrow$ Pn$\bar{3}$m transition, indicating strong coupling between protein accommodation and mesophase structure	Valldeperas et al. (2019b)

## Biophysical consequences of enzyme nanoconfinement

In the previous sections, we discussed how lipidic mesophases organize water, lipids, and enzymes at the nanoscale. The next question is how this organization may affect enzymatic structure and catalytic behavior. The evidence considered in this section originates from both bulk lipidic mesophases and dispersed LCNPs, and the experimental format is explicitly identified where relevant. In both classes of systems, catalytic performance depends strongly on two interrelated factors: **the geometry of the internal aqueous network**, which controls molecular transport, and the **enzyme–lipid interface**, which modulates localization, stability, and conformational dynamics. In dispersed LCNPs, however, additional contributions from finite particle dimensions, colloidal interfaces, enzyme leakage, surface adsorption, and exchange with the surrounding continuous phase must also be considered.

The most quantitative structure–function relationships reported for enzymes confined in lipidic mesophases are summarized in Table 1. The available studies indicate that aqueous channel diameter and network topology primarily control mass transport, lag time, and apparent kinetic parameters, whereas enzyme–lipid compatibility becomes particularly important for conformational and operational stability.

### Transport, diffusion, and microenvironment effects

The confinement of enzymes within the aqueous channels of lipid mesophases introduces substrate and product transport restrictions that do not exist in homogeneous solution. In nanometric channels, diffusion is geometrically constrained by the lipid bilayer walls, increasing the effective pathway traversed by the substrate and reducing its rate of arrival at the active site. The immediate kinetic consequence is often an increase in the apparent $K_M$, which incorporates diffusional resistance in addition to the intrinsic enzyme–substrate affinity (Gilbert et al. 2019).

A second indicator of diffusional limitation is the lag time, defined as the initial period of low activity observed before reaching steady state. Lag time increases as the channel diameter decreases (Sun et al. 2014). Both phenomena can be integrated by the effectiveness factor η, defined as the ratio between the activity observed in meso and the intrinsic activity of the free enzyme in the absence of diffusional limitations. For β-galactosidase confined in $L_3$-NPs, $\eta \approx 0.5$, indicating that only half of the activity is effectively accessible under the assay conditions (Gilbert et al. 2019).

Mendozza and co-workers and Sun and co-workers conducted systematic studies relating the aqueous channel diameter $D_W$ to the magnitude of diffusional constraint. Mendozza et al. (2020) explicitly separated the contributions of substrate and enzyme confinement in bulk cubic phases of phytantriol swollen with sucrose stearate, with $D_W$ tunable from 2.3 to 3.6 nm. When alkaline phosphatase was held external to the mesophase and its substrate p-nitrophenyl phosphate was confined within the aqueous channels, the conversion rate scaled linearly with $D_W$. It was approximately 31-fold lower than in free solution at $D_W = 2.3$ nm, and 14-fold lower at $D_W = 3.6$ nm. When the enzyme itself was confined within the channels, the kinetics were instead dominated by a lag period that shortened progressively with increasing $D_W$, consistent with restricted diffusion of the substrate to the catalytic centers (Mendozza et al. 2020). A similar trend was reported by Sun and co-workers, who swelled a Pn$\bar{3}$m cubic phase of monoglyceride with sugar ester to achieve $D_W$ values ranging from 3.5 to 7.2 nm. The lag time of physically entrapped horseradish peroxidase decreased from 1260 s at $D_W = 3.5$ nm to 119 s at $D_W = 7.2$ nm. As $D_W$ increased, the apparent Michaelis constant dropped from 7.5 mM to 3.0 mM, approaching the free-solution value of 1.4 mM as diffusional restriction was progressively relieved (Sun et al. 2014). Together, these studies establish $D_W$ as a fundamental structural parameter for modulating in meso enzymatic activity.

When the channel diameter is held constant, the topology of the aqueous network becomes an independent determinant of enzymatic activity. In a controlled experiment with HRP in phases sharing the same $D_W$, approximately 3.4 nm, but different space groups, relative activity followed the sequence Pn$\bar{3}$m > Ia$\bar{3}$d > $H_{II}$, with values of 100%, 38.5%, and 15.1%, respectively (Sun et al. 2015). This hierarchy reflects the connectivity of the aqueous network (Fig. 3D). The Pn$\bar{3}$m phase possesses four-fold channel junctions, allowing more isotropic three-dimensional diffusion. The Ia$\bar{3}$d phase features three-fold junctions, imposing a more restricted three-dimensional pathway. In turn, the $H_{II}$ phase contains unbranched (i.e. non-connected water channels), one-dimensional cylindrical channels, determining the longest and least flexible trajectory. The linear diffusive behavior observed as a function of the square root of time confirmed that activity was limited by mass transport rather than by the intrinsic reaction rate (Sun et al. 2015).

The sponge phase $L_3$ provides a particularly useful example in which results from bulk and dispersed systems can be compared. In bulk $L_3$ mesophases, the absence of crystalline periodicity and the presence of relatively large and flexible aqueous domains reduce some of the strict geometric restrictions characteristic of $Q_{II}$ and $H_{II}$ phases. In dispersed $L_3$ nanoparticles, the same internal sponge-like organization is combined with a finite particle boundary and an external colloidal interface, introducing additional possibilities for enzyme exchange, leakage, and surface association.

In this case, the most significant effects often arise from the interfacial microenvironment. Raman spectroscopy revealed changes in Tyr and Trp bands of aspartic protease confined in lipid LLC, consistent with a more hydrophobic and less hydrated environment around the enzyme compared with solution (Valldeperas et al. 2019b). In the corresponding sponge-phase dispersions, the confined protease exhibited higher activity than the free enzyme at the same temperature, suggesting that the lipid interface may stabilize catalytically favorable conformations or modify the hydration state around the active site. In dispersed $L_3$-NPs loaded with β-galactosidase, the apparent $K_M$ increased from 1.9 to 2.9 mM, attributed primarily to diffusion limitation of the ONPG substrate through the sponge-phase channels, with a secondary contribution from restricted active-site accessibility due to enzyme orientation at the lipid–water interface (Gilbert et al. 2019).

Water confined within lipid mesophases also exhibits dynamics and electrostatic properties distinct from bulk water. Ultrafast IR spectroscopy further shows that interfacial water in lipid mesophases reorients ${\sim }2$-fold more slowly than core water, with the dynamic gap between both populations narrowing as interfacial curvature increases from planar ($L_\alpha$) to bicontinuous (Ia$\bar{3}$d) geometry (Catalini et al. 2026). Terahertz absorption spectroscopy combined with atomistic molecular dynamics simulations has revealed interfacial and bulk-like water populations, both with increased reorientation times and perturbation of the hydrogen-bonding network extending up to approximately 2.4 nm from the membrane midplane (Zunzunegui-Bru et al. 2025). Additional theoretical studies on nanoconfinement suggest significant reductions in the local dielectric constant relative to bulk water (Mondal and Bagchi 2024). This altered environment should be considered when interpreting kinetic and stability data, since ionization states of catalytic residues and electrostatic interactions at the enzyme–lipid interface are sensitive to the local dielectric environment.

### Stabilization and functional modulation

Confinement within lipidic mesophases and LCNPs can impart measurable stability gains to encapsulated enzymes. These gains may involve storage stability, operational stability, proteolytic protection, and conformational stability. The underlying mechanisms include restricted conformational mobility, maintenance of a hydrated microenvironment, reduced exposure to denaturing interfaces, physical protection from macromolecular degradation agents, and favorable enzyme–lipid interactions.

For β-galactosidase confined in $L_3$ nanoparticles, the first-order deactivation constant was $3.4 \times 10^{-6}$ s$^{-1}$, lower than that measured for the free enzyme at room temperature, $5.1 \times 10^{-6}$ s$^{-1}$. Activity remained detectable after 12 days of incubation, and 100% encapsulation efficiency was maintained for 5 months (Gilbert et al. 2019). Stability gains were also observed for aspartic protease in bulk sponge phase over 6 days (Valldeperas et al. 2019b) and for catalase in lipid cubosomes for 90 days without detectable structural degradation (Rakotoarisoa et al. 2019). In these cases, stabilization has been associated with the ability of the lipidic matrix to restrict large-amplitude conformational fluctuations, thereby limiting structural rearrangements associated with unfolding, subunit dissociation, or progressive denaturation.

Physical protection against proteolytic degradation is another important stabilization mechanism. In this case, the lipid matrix acts as a barrier that limits access of exogenous proteases to cleavage sites on the encapsulated enzyme. Monoolein and phytantriol LCNPs loaded with the glycoside hydrolase PslG preserved antibiofilm activity after incubation with proteinase K, whereas the free enzyme was completely inactivated under the same conditions (Thorn et al. 2021). The high encapsulation efficiency reported for monoolein and phytantriol LCNPs, 92–94%, is consistent with the observed protection, since the internalized fraction is expected to remain less accessible to proteolytic attack, while surface-associated enzyme may still be degraded. This example illustrates one of the most advanced functional applications of enzyme-loaded LCNPs, as protection from proteolysis directly translates into improved biological performance in contexts relevant to therapeutic enzyme delivery.

However, stabilization is not universal and cannot be predicted solely from encapsulation efficiency. The functional outcome depends strongly on the compatibility between the enzyme surface and the lipid microenvironment. Membrane-associated or interfacial enzymes, whose native function already depends on lipid–water interfaces, may benefit from confinement more readily than highly hydrophilic enzymes. This distinction was illustrated in a comparative study of D-fructose dehydrogenase, a membrane-associated enzyme of approximately 140 kDa, and horseradish peroxidase, a soluble hydrophilic enzyme of approximately 40 kDa, incorporated into the same Pn$\bar{3}$m monoolein phase. D-fructose dehydrogenase retained 100% of its activity after 5 days of incubation and 63% after 10 reuse cycles, whereas horseradish peroxidase retained only 21% activity under comparable conditions (Sun et al. 2016). The apparent $K_M$ of D-fructose dehydrogenase in meso, 1.7 mM, remained close to that measured in solution, 1.3 mM. In contrast, the apparent $K_M$ of horseradish peroxidase increased from 1.4 to 7.5 mM, indicating that confinement imposed functional restrictions on substrate accessibility or enzyme dynamics.

**Fig. 4:**
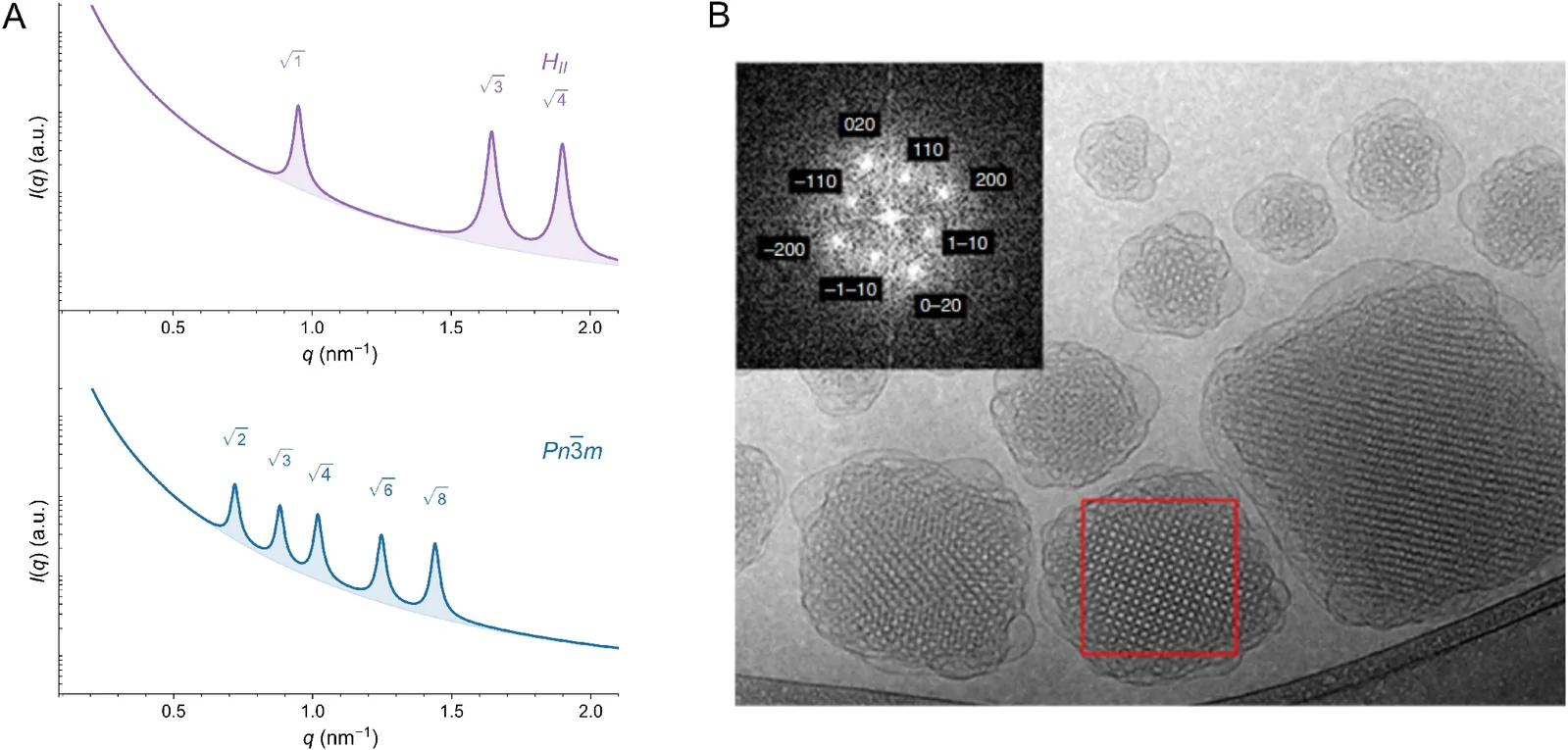
Structural characterization of LCNPs by SAXS and cryo-TEM. **A** Representative SAXS profiles of $H_{II}$ (upper) and Pn$\bar{3}$m (lower) phases with characteristic Bragg peak ratios indicated. **B** Cryo-TEM image of cubosomes and fast Fourier transform (FFT, inset) of the boxed particle, with indexed reflections confirming cubic symmetry. Reproduced from Demurtas et al. (2015) under a Creative Commons Attribution 4.0 International License (CC BY 4.0)

These observations show that lipidic mesophases can either preserve or perturb catalytic function depending on the enzyme–lipid pair. Favorable confinement may stabilize the active conformation, catalytic site. Unfavorable confinement may restrict substrate diffusion, alter active-site accessibility, or promote non-productive adsorption at the lipid interface or stabilizer corona. Therefore, stability gains should always be evaluated together with functional readouts, including residual activity, apparent kinetic parameters, reuse cycles, leakage, protease resistance, and structural integrity.

## Characterization of enzyme–mesophase and enzyme–LCNP systems

### Structural characterization

Small-angle X-ray scattering (SAXS) is the reference technique for structural characterization of lipid mesophases and LCNPs, providing two central parameters for describing the enzymatic microenvironment: the symmetry of the mesophase and its lattice parameter, *a*. In periodic mesophases, structural assignment is based on the characteristic sequence of relative Bragg peak positions associated with the allowed reflections of each lattice. Representative SAXS patterns of the inverse hexagonal $H_{II}$ and bicontinuous cubic Pn$\bar{3}$m phases are shown in Fig. 4A. A detailed description of the principles and practical indexing of lyotropic liquid-crystalline mesophases can be found in the classical treatment by Hyde (Hyde 2001). Once the mesophase symmetry has been identified, the lattice parameter *a* is calculated from the indexed Bragg reflections. The aqueous channel diameter, $D_W$, is not obtained directly from the SAXS profile, but is derived from *a* using symmetry-dependent geometrical relationships specific to each mesophase architecture (Mariani et al. 1988; Caselli et al. 2024; Sarkar et al. 2019).

The sponge phase $L_3$ represents a particular case, as it lacks long-range periodicity and produces diffuse peaks rather than Bragg peaks, allowing only estimation of the characteristic scale of the aqueous channels (Valldeperas et al. 2019a). Variations induced by enzyme loading are obtained directly from SAXS profiles and constitute one of the primary structural indicators of interaction between the enzyme and the mesophase.

Enzyme loading frequently alters the lattice parameter, and this variation has been interpreted as evidence of incorporation into the mesophase. The magnitude of Δa depends on the architecture of the protein, particularly the volume of hydrophilic domains projected into the aqueous space of the channels. In a study with the phosphoethanolamine transferase NmEptA in a Pn$\bar{3}$m phase of monoolein, an increase in *a* from 105 to 120 Å was statistically significant and attributed to the hydrophilic domain of the enzyme, whose dimensions exceed the aqueous channel diameter and impose expansion of the aqueous network (van’t Hag et al. 2017). In contrast, Na$^+$/K$^+$-ATPase in the same Pn$\bar{3}$m phase did not produce a statistically significant change in *a*, consistent with a transmembrane protein lacking a bulky hydrophilic domain projected into the channels (Zatloukalova et al. 2018). In hexosomes loaded with HMG-CoA reductase, *a* varied from 5.5 to 5.9 nm, an expansion attributed to the hydrophilic catalytic domain that widens the cylindrical aqueous channel of the $H_{II}$ phase (Zaborowska-Mazurkiewicz et al. 2025). Thus, Δa can indicate occupation of the aqueous space by the protein, but its absence does not exclude reconstitution of the enzyme within the lipid bilayer. Cryogenic transmission electron microscopy complements SAXS by providing structural information at the single-particle level. The technique preserves LCNPs in the hydrated state, which allows direct visualization of the internal mesophase organization.

Figure 4B shows a representative cryo-TEM micrograph of phytantriol cubosomes, together with the fast Fourier transform (FFT) of the highlighted region and assignment of the principal reflections (Demurtas et al. 2015). An Im$\bar{3}$m cubosome may display square, hexagonal, or rhombic arrangements depending on its orientation relative to the electron beam. Thus, quantitative analysis can be performed by an FFT analysis of selected regions, generating a power spectrum with peaks at *q* positions corresponding to families of *hkl* planes. Unlike SAXS, which provides ensemble-averaged information, cryo-TEM reveals structural heterogeneities and minor particle populations. This feature allowed identification of coexisting sponge-phase nanoparticles and unilamellar vesicles within the same formulation (Donida et al. 2018). For in situ characterization in cellular environments, cryo-SIM combined with cryo-SXT has been used to localize internalized LCNPs and evaluate preservation of internal structure. Ribeiro and co-workers localized Pn$\bar{3}$m and Im$\bar{3}$m cubosomes within lysosomes of HeLa cells, demonstrating maintenance of internal organization after cellular internalization (Ribeiro et al. 2025).

Dynamic light scattering and zeta potential are complementary tools for evaluating size, polydispersity, and colloidal stability after enzyme loading. DLS allows detection of particle aggregation or destabilization that may not be evident in SAXS profiles. Changes in zeta potential provide an indirect indicator of enzyme localization, as adsorption onto the external surface tends to modify particle charge more strongly than encapsulation within internal channels. In hexosomes loaded with HMG-CoA reductase, for instance, the zeta potential shifted from -19 to -7 mV, suggesting interaction of the enzyme with the particle surface without compromising colloidal stability (Zaborowska-Mazurkiewicz et al. 2025).

### Conformational characterization

Spectroscopic techniques are used to investigate the conformational state of confined enzymes and provide clues regarding localization. Infrared and Raman spectroscopies have been applied to confirm the preservation of the secondary structure in the cubic phases and bulk LCNPs (Razumas et al. 1997; Valldeperas et al. 2019b; Astolfi et al. 2021). More recently, solid-state nuclear magnetic resonance was used to investigate the localization and dynamics of an integral membrane enzyme in a phase of Pn$\bar{3}$m, revealing simultaneous distribution in both bilayer and aqueous channels (Ramberg et al. 2025).

Circular dichroism is the reference technique for quantitative determination of protein secondary structure, although its application in lipid mesophases depends on the optical properties of the system. In a cubic phase of lysophosphatidylcholine, a lipid transparent in the far-UV region, Portmann et al. achieved CD spectra of chymotrypsin compatible with its native structure (Portmann et al. 1991). In monoolein phases, lipid absorption below approximately 215 nm prevents acquisition of far-UV spectra using conventional equipment. Under this condition, only the near-UV region remains accessible, providing information about the microenvironment of aromatic residues but not global secondary structure (Li and Caffrey 2020). In phytantriol phases, this limitation can be overcome using synchrotron radiation. Strachan and co-workers showed via SR-CD that the alpha-helical structure of a protein encapsulated in Pn$\bar{3}$m phytantriol was preserved against proteolytic degradation, whereas the free protein was completely degraded under the same conditions (Strachan et al. 2021). In dispersed LCNPs, light scattering of the colloidal suspension represents an additional limitation, as reflected by the scarcity of CD studies in dispersed enzymatic systems.

Intrinsic tryptophan fluorescence provides information about the microenvironment near the polypeptide chain. The emission maximum reflects the polarity around Trp residues, although the orientation of electric dipoles near the indole ring can influence emission independently of medium polarity (Vivian and Callis 2001; Raw et al. 2023). Mishraki and co-workers applied this approach to lysozyme confined in a bulk inverse hexagonal phase of monoolein, inferring the positioning of Trp residues at the lipid interface (Mishraki et al. 2010). Fluorescence quenching by agents that partition into the bilayer provides complementary information about protein localization within the mesophase. Li and Caffrey used this strategy to confirm reconstitution of an integral membrane enzyme into the lipid bilayer of a monoolein cubic phase (Li and Caffrey 2011).

### Functional characterization

Functional characterization is essential to determine whether enzyme confinement in lipidic mesophases or LCNPs preserves, enhances, or restricts catalytic performance. In these systems, apparent activity reflects a combination of enzyme loading, localization, conformational preservation, substrate partitioning, product diffusion, and mass transport through aqueous or interfacial domains. Functional assays should therefore be interpreted together with structural information, particularly the mesophase space group, lattice parameter, aqueous channel diameter, and possible structural transitions induced by enzyme loading or substrate conversion.

Spectrophotometric assays are the most widely used methods to quantify enzymatic activity in mesophases and LCNPs, especially when substrates or products display defined optical absorption. Portmann and co-workers monitored α-chymotrypsin encapsulated in a lysophosphatidylcholine cubic phase using Suc-Ala-Phe-pNA as substrate and followed p-nitroaniline release by absorbance at 410 nm (Portmann et al. 1991). The authors observed an approximately 2 h lag phase, attributed to the time required for substrate diffusion through the mesophase channels. In phytantriol cubic phases with aqueous channel diameter modulated by sucrose stearate, alkaline phosphatase activity was quantified by p-nitrophenol formation, revealing a linear relationship between $D_W$ and reaction rate (Mendozza et al. 2020). β-Galactosidase encapsulated in sponge-phase nanoparticles retained activity toward ONPG, although the apparent $K_M$ increased from 1.9 mM in solution to 2.9 mM in $L_3$-NPs (Gilbert et al. 2019). For HMG-CoA reductase encapsulated in hexosomes, activity was monitored by NADPH consumption at 340 nm, resulting in 2.23 units mg$^{-1}$ in hexosomes compared with 0.91 units mg$^{-1}$ in solution (Zaborowska-Mazurkiewicz et al. 2025).

Electrochemical assays provide a complementary approach to evaluate catalytic activity, substrate responsiveness, and inhibitor accessibility. Zatloukalova and co-workers immobilized Na$^+$/K$^+$-ATPase in a Pn$\bar{3}$m monoolein phase on a carbon electrode and monitored ATP hydrolysis through inorganic phosphate formation, detected by square-wave voltammetry and by a spectrophotometric phosphomolybdate assay. The immobilized enzyme displayed an activity of 4.62 nmol P$_\textrm{i}$ mg$^{-1}$ min$^{-1}$ and remained stable for 7 days, whereas the enzyme in solution lost activity within 1–4 days depending on the preparation protocol (Zatloukalova et al. 2018). Inhibition by ouabain confirmed that the activity was specific to Na$^+$/K$^+$-ATPase and demonstrated that the inhibitor could access the active site through the aqueous network. For HMG-CoA reductase encapsulated in hexosomes, activity was confirmed by cyclic voltammetry in the presence of ABTS, and fluvastatin produced 95–98% inhibition, indicating that hexosomal confinement did not compromise drug accessibility to the active site (Zaborowska-Mazurkiewicz et al. 2025).

For enzymes acting within the lipid bilayer or generating products without convenient optical signatures, chromatographic and mass spectrometric methods provide the required analytical specificity. Van’t Hag and co-workers investigated the activity of the phosphoethanolamine transferase NmEptA in a Pn$\bar{3}$m phase by monitoring the conversion of DOPE into DOG and MOPE (van’t Hag et al. 2017). Product formation was quantified by RP-HPLC, and product identity was confirmed by ESI-TOF MS. Conversion reached 90% after 24 h, with an estimated rate of approximately 1 μM s$^{-1}$. In the same study, the variation in the lattice parameter of the Pn$\bar{3}$m phase was proportional to the fraction of DOPE converted, allowing catalytic progress to be monitored in real time by SAXS. This result demonstrates that, in lipidic mesophases, the reaction medium itself can become a structural reporter of enzymatic activity.

Coupled enzymatic assays are useful when direct detection of products is not straightforward. For ADP-producing enzymes such as diacylglycerol kinase and phosphatidylglycerol synthase, Li and Caffrey developed assays based on the pyruvate kinase/lactate dehydrogenase system, in which ADP release is coupled to NADH depletion at 340 nm. Using this approach, they determined a $K_M$ of 3.5 mM for Mg$^{2+}$-ATP and a specific activity of 14.4 U mg$^{-1}$ for diacylglycerol kinase in monoolein lipidic cubic phase (Li and Caffrey 2011, 2020). Coupled assays expand the range of detectable reactions, but the coupling enzymes, cofactors, and auxiliary substrates must themselves remain compatible with the mesophase environment and must not become the rate-limiting components of the assay.

The choice of functional assay should be guided by the chemical nature of the reaction, expected enzyme localization, and physicochemical properties of substrates and products. Hydrophilic substrates may be controlled primarily by diffusion through aqueous channels, whereas hydrophobic or amphiphilic substrates may partition into the lipid bilayer and accumulate near interfacial enzymes. Redox enzymes require careful evaluation of cofactor or mediator accessibility, while membrane-associated enzymes may demand analytical methods capable of resolving lipid substrates and products. In all cases, apparent kinetic parameters such as $K_M$, $V_\textrm{max}$, $k_\textrm{cat}$, and specific activity should be interpreted as system-level parameters that reflect both enzyme catalysis and mesophase-imposed transport or partitioning effects.

## Functional perspectives and emerging applications

Beyond describing how mesophases affect enzyme structure and kinetics, it is important to distinguish applications demonstrated in bulk lipidic mesophases from those realized using dispersed LCNPs. Bulk mesophases have been particularly valuable as nanostructured reaction media, membrane-mimetic matrices, and bioelectrochemical films, whereas dispersed cubosomes, hexosomes, and sponge-phase nanoparticles offer additional opportunities as colloidal enzyme carriers for delivery, protection, transport, and operation in biological media. These two formats therefore address related but distinct technological questions. Their nanometric aqueous networks and lipid–water interfaces enable control over substrate partitioning and enzyme microenvironment in both cases, but dispersed LCNPs additionally require consideration of colloidal stability, enzyme retention or leakage, external surface adsorption, and interactions with the surrounding medium. The representative applications summarized below are therefore identified explicitly as either bulk or dispersed systems.

Representative functional implementations of enzyme-containing lipidic mesophases and LCNPs are summarized in Table 2. The examples illustrate the transition from fundamental studies of enzyme confinement toward biosensing, inhibitor screening, membrane-enzyme assays, therapeutic enzyme delivery, enzymatic cascades, and biocatalytic microreactors.

**Table 2:** Representative functional applications of enzymes confined in bulk lipidic mesophases and dispersed lipid liquid-crystalline nanoparticles (LCNPs). The distinction between bulk and dispersed systems is explicitly indicated because transport, colloidal behavior, enzyme retention, and practical implementation differ substantially between these platforms

Platform	Mesophase/system	Enzyme(s)	Application	Representative functional outcome	Ref.
Bulk mesophase on electrode	Monoolein Pn$\bar{3}$m	Glucose oxidase, pyranose oxidase, and laccase	Electrochemical biosensing	Catalytic activity and substrate responsiveness were maintained in the cubic phase. Approximately 90% activity retention after seven days was reported for the oxidase-based systems	Nazaruk and Bilewicz (2007)
Bulk mesophase on electrode	Lipidic cubic phase with carbon nanotubes	D-Fructose dehydrogenase and laccase	Direct electron transfer and enzymatic biofuel cell	Direct electron transfer from fructose dehydrogenase generated a current density of 3.2 mA cm$^{-2}$. The corresponding biofuel cell reached an open-circuit potential of 703 mV and a maximum power output of 860 μW cm$^{-2}$	Nazaruk et al. (2014)
Bulk mesophase on electrode	Monoolein cubic phase containing Fc12	Glucose oxidase	Redox-mediated biosensing	The amphiphilic mediator was retained at the lipid–water interface. The GOx-loaded electrode followed Michaelis–Menten kinetics toward glucose, with an apparent $K_M$ of 8.9 mM and a linear response between 2 and 17 mM	Liu et al. (2024)
Bulk mesophase on electrode	Monoolein Pn$\bar{3}$m	Na$^+$/K$^+$-ATPase	Membrane-enzyme assay and inhibitor screening	ATPase activity and electrogenic ion transport remained detectable in meso. Both responses were sensitive to ouabain, demonstrating accessibility of the pharmacological target within the cubic phase	Zatloukalova et al. (2018, 2019)
Dispersed LCNPs	$H_{II}$ hexosomes	HMG-CoA reductase	Drug-target and inhibitor screening	Confined enzyme activity reached 2.23 units mg$^{-1}$ compared with 0.91 units mg$^{-1}$ in solution. Fluvastatin produced 95–98% inhibition, demonstrating inhibitor accessibility within the nanoparticle system	Zaborowska-Mazurkiewicz et al. (2025)
Bulk mesophase	Monoolein Pn$\bar{3}$m	NmEptA	Membrane-enzyme catalysis and structural activity readout	Approximately 90% conversion of DOPE was achieved after 24 h. Catalytic progress correlated with changes in the cubic lattice parameter measured by SAXS, allowing the mesophase itself to act as a structural reporter of enzymatic activity	van’t Hag et al. (2017)
Bulk mesophase	Lipidic cubic phase	Glucose oxidase and horseradish peroxidase	Enzymatic cascade and biosensing	The two enzymes operated sequentially within the mesophase, demonstrating the feasibility of coupled enzymatic reactions while preserving the cubic architecture	Vallooran et al. (2016)
Dispersed LCNPs	Monoolein- and phytantriol-based LCNPs	PslG	Protective enzyme delivery and antibiofilm therapy	LCNP confinement protected PslG against proteinase K and enabled lipase-responsive release while preserving antibiofilm activity. This system illustrates the use of dispersed LCNPs as protective enzyme-delivery platforms	Thorn et al. (2021)
Dispersed cubosomes	Pn$\bar{3}$m/Im$\bar{3}$m cubosomes	Catalase	Intracellular enzyme delivery	Catalase loading modified the internal cubic structure, and treatment with enzyme-containing cubosomes produced an approximately 1.5-fold increase in intracellular peroxidatic activity in differentiated SH-SY5Y cells	Rakotoarisoa et al. (2019)

### Bioelectrochemical devices and biosensors

When deposited onto electrode surfaces, lipidic cubic phases create hydrated nanostructured films in which confined enzymes react with diffusible substrates while redox mediators or conductive additives transfer electrons to the electrode. Glucose oxidase, pyranose oxidase, and laccase were among the first redox enzymes studied in this configuration using a Pn$\bar{3}$m monoolein phase on Pt and glassy carbon electrodes (Nazaruk and Bilewicz 2007). Kinetic parameters were preserved by confinement, with $K_M$ values of 5.3 mM for glucose oxidase and 4.5 mM for pyranose oxidase in the absence of a mediator, and approximately 90% activity retention after seven days at room temperature.

The high mobility of redox mediators within the aqueous channels is a key feature of these systems. The [Ru(NH$_3$)$_6$]$^{3+}$ complex diffuses at $2.2 \times 10^{-6}$ cm$^2$ s$^{-1}$ in monoolein LCP, approximately $10^4$ times faster than in Nafion films, a polymer widely used as a benchmark matrix in enzymatic electrodes (Nazaruk and Bilewicz 2007; Nazaruk et al. 2008). This permeability is accompanied by an increase in the capacitance of the electrode–solution interface after LCP deposition on Pt, from 9.6 to 22.4 μF cm$^{-2}$, consistent with a continuous aqueous network connecting the bulk solution to the electrode surface.

Subsequent refinements aimed to reduce dependence on soluble mediators, which can diffuse out of the film over time. Incorporation of single-walled carbon nanotubes into LCP enabled direct electron transfer from fructose dehydrogenase to the electrode, producing a current density of 3.2 mA cm$^{-2}$ and a $K_M$ of 12 mM for fructose (Nazaruk et al. 2014). A biofuel cell constructed in this configuration, with laccase as the biocathode enzyme, reached an open-circuit potential of 703 mV and a maximum power output of 860 μW cm$^{-2}$. More recently, an amphiphilic redox mediator, Fc12, was anchored into the bilayer of monoolein LCP, positioning the redox-active group at the interface with the aqueous channels where glucose oxidase was confined (Liu et al. 2024).

### In meso assays for membrane enzymes and drug targets

Lipidic mesophases are also useful for functional characterization of membrane enzymes and pharmacological targets. Na$^+$/K$^+$-ATPase incorporated into the Pn$\bar{3}$m phase of monoolein responded in a dose-dependent manner to ouabain, with suppression of the electrochemical signal proportional to inhibitor concentration (Zatloukalova et al. 2018, 2019). The same platform enabled detection of current generated by Na$^+$ transport, dependent on Na$^+$ and K$^+$ concentrations and suppressed by ouabain, indicating that both ATPase and ion-pumping activities remained accessible in meso.

HMG-CoA reductase, the target of statins, has been incorporated into hexosomes and shown to retain activity and responsiveness to inhibitors detectable by cyclic voltammetry (Zaborowska-Mazurkiewicz et al. 2025). Likewise, the phosphoethanolamine transferase NmEptA, which contributes to bacterial resistance to colistin by modifying lipopolysaccharides, maintained activity in LCP under conditions compatible with 96-well screening assays. In this case, catalytic progress was monitored by changes in the lattice parameter measured by SAXS (van’t Hag et al. 2017). The kinetic parameters of diacylglycerol kinase DgkA were also determined directly in meso, confirming the compatibility of LCP with catalytic assays for lipid-processing membrane proteins (Li and Caffrey 2020). More recently, solid-state NMR was used to study the peptidase LspA, a target of the antibiotic globomycin, incorporated into a Pn$\bar{3}$m monoolein phase. The study confirmed simultaneous distribution of the enzyme in both the bilayer and aqueous channels and revealed an inhibitor-induced conformational rearrangement (Ramberg et al. 2025).

### Enzymatic cascades and soft-matter microreactors

Although cascade enzymatic catalysis is frequently cited as a potential application of nanoconfinement, its realization in dispersed LCNPs remains largely unexplored. Available examples are currently restricted to bulk phases. In monoolein LCP, glucose oxidase and horseradish peroxidase were co-encapsulated and acted sequentially to generate a birefringence detection signal proportional to glucose concentration while preserving the cubic structure during the reaction (Vallooran et al. 2016). In lamellar phytantriol mesophases, enzymatic reactions were demonstrated at subzero temperatures. Isolated HRP oxidized tetramethylbenzidine, producing a product stable for weeks at $-20~^\circ$C. In the GOx–HRP cascade, GOx converted glucose into H$_2$O$_2$, subsequently used by HRP to oxidize TMB. The signal measured at $-20~^\circ$C was higher than that observed at 25 $^\circ$C and 4 $^\circ$C (Yao et al. 2021).

Extending this principle to dispersed cubosomes, hexosomes, and sponge-phase nanoparticles represents one of the clearest opportunities in the field. The compartmentalized aqueous networks and lipid–water interfaces of LCNPs could, in principle, organize sequential reactions, improve local intermediate transfer, protect labile enzymes, and enable catalysis under conditions that are challenging for free enzymes. The field itself anticipated this possibility by recognizing that cubic phases would be suitable for sequential reactions associated with lipid metabolic pathways (Li and Caffrey 2011). However, systematic demonstrations of multienzymatic catalysis in dispersed LCNPs are still lacking.

### Molecular bioeconomy and biorefinery opportunities

The connection between enzyme-loaded LCNPs and molecular bioeconomy remains emerging but conceptually important. The most obvious biorefinery enzymes include cellulases, xylanases, lipases, laccases, sugar isomerases, oxidoreductases, and enzymes involved in lipid or carbohydrate upgrading. However, the current literature is highly uneven. Lipases and laccases provide the most direct connections to lipid transformation, interfacial catalysis, and oxidative bioelectrochemistry in lipidic mesophases, whereas direct examples involving cellulases, xylanases, or xylose isomerase in cubosomes, hexosomes, or sponge-phase nanoparticles remain scarce or absent.

This absence should not be interpreted as a lack of relevance, but rather as an opportunity for new discoveries in this field. Classical biomass-degrading enzymes such as cellulases and xylanases act on insoluble polymeric substrates, which may be poorly compatible with narrow aqueous channels of many LCNPs. For these enzymes, LCNPs may not replace established immobilization supports for bulk biomass hydrolysis. Instead, their potential may lie in specific steps where nanoscale organization, interfacial catalysis, or substrate partitioning are decisive. Examples include lipase-mediated transformations of oils and fatty acids, laccase-mediated oxidation of phenolics and lignin-derived molecules, redox biocatalysis involving hydrophobic substrates, and enzymatic synthesis of carbohydrate-derived building blocks. It should be stressed that Brazil plays a key role in this field and still lacks the development of such platforms for biomass-degrading enzymes complexed with nanoparticles.

The enhanced activity and stability of D-fructose-6-phosphate aldolase confined in lipidic cubic mesophases for asymmetric carbohydrate synthesis illustrates how lipidic nanoconfinement can contribute to renewable carbon valorization beyond classical hydrolysis reactions (Zhou et al. 2018). From this perspective, LCNPs may emerge as specialized soft-matter microreactors for selected steps in biorefinery cascades, particularly those requiring hydration control, interfacial organization, substrate partitioning, or compatibility with hydrophobic and amphiphilic molecules. This direction is especially relevant for biomass-rich and biodiversity-rich contexts, in which enzyme-enabled upgrading of oils, lipids, carbohydrates, and lignin-derived molecules is central to the development of a molecular bioeconomy.

## Conclusions and outlook

This review addressed enzymatic confinement in lipid mesophases from three complementary perspectives. From the standpoint of structural diversity, bicontinuous cubic, inverse hexagonal, and sponge phases offer aqueous microenvironments with channel diameters ranging from approximately 2 to 13 nm. These diameters are tunable by lipid composition, measurable by SAXS, and partially preserved when the system is dispersed into cubosomes, hexosomes, and sponge-phase nanoparticles. From the standpoint of catalytic behavior, confinement can preserve activity, impose diffusional restrictions, alter apparent kinetic parameters, stabilize enzymes against degradation, and modulate function according to channel geometry, topology, hydration, and enzyme–lipid affinity. From the characterization standpoint, the combination of SAXS, cryo-TEM, DLS, spectroscopies, localization assays, and activity measurements is essential to establish structure–function relationships in these systems.

Overall, the available literature supports a two-axis framework for understanding enzyme behavior in lipidic mesophases. The first axis is geometric and is defined by aqueous channel diameter and network topology. This axis governs substrate diffusion, product removal, lag time, apparent $K_M$, and effectiveness factor. The second axis is interfacial and is defined by the affinity between the enzyme surface and the lipid bilayer. This axis governs enzyme localization, conformational stability, operational stability, and compatibility with hydrophobic or amphiphilic substrates. The functional outcome of enzyme confinement results from the interplay between these two axes. Therefore, lipidic mesophases should not be treated as generic immobilization supports, but as tunable catalytic microenvironments whose structure must be matched to the enzyme, substrate, and intended application.

The growth of the literature in this field since 2020 allows it to be situated relative to previous synthesis works. Mezzenga and co-workers consolidated the use of lipid mesophases as nanostructured matrices in a broad context, but without emphasis on enzymatic behavior (Mezzenga et al. 2019). Manni and co-workers explicitly identified the absence of studies on enzymatic confinement in lipid cubosomes as an important gap in the field (Manni et al. 2020). Since then, studies have begun to demonstrate functional encapsulation of enzymes in cubosomes, hexosomes, and sponge-phase nanoparticles, combining SAXS, cryo-TEM, spectroscopies, and activity assays. The present review synthesizes this progress and proposes that the relationship between internal LCNP geometry and enzymatic behavior requires integrated characterization approaches.

Despite these advances, several frontiers remain open. Cascade enzymatic catalysis in dispersed LCNPs is still scarce, contrasting with progress achieved in bulk lipid phases and in other confinement systems, such as metal–organic frameworks (Chen et al. 2018) and polymersomes (Vriezema et al. 2007; Peters et al. 2014). Standardized BioSAXS and functional assay protocols for enzyme-loaded LCNPs are still lacking, limiting direct comparison between studies. The extension of catalysis to extreme conditions, including subzero temperatures, remains largely unexplored in dispersed cubosomes and hexosomes. Finally, the application of LCNPs to molecular bioeconomy and biorefinery reactions is still at an early stage, especially for enzymes involved in biomass deconstruction, lipid upgrading, lignin-derived transformations, and renewable carbon valorization.

The inclusion of this review in the celebratory edition of the 90th anniversary of the SBBf also reflects the growth of national research on lipidic nanostructures and biomolecular delivery. Brazilian groups have contributed to the development of LCNPs as carriers for drugs and therapeutic biomolecules, including pH-responsive cubosomes for oral delivery (Mertins et al. 2020), monoolein nanoparticles for delivering enzymes applied to lysosomal storage diseases (Donida et al. 2018), and super-resolution microscopy combined with cryogenic X-ray tomography for intracellular localization of cubosomes (Ribeiro et al. 2025). Building on this foundation, enzyme-loaded LCNPs offer an opportunity to connect soft-matter biophysics, enzymology, applied biocatalysis, and molecular bioeconomy.

## Data Availability

No datasets were generated or analysed during the current study.

## References

[CR1] Angelova A, Angelov B, Mutafchieva R, Lesieur S, Couvreur P (2011) Self-assembled multicompartment liquid crystalline lipid carriers for protein, peptide, and nucleic acid drug delivery. Acc Chem Res 44(2):147–156. 10.1021/ar100120v 21189042

[CR2] Astolfi P, Giorgini E, Perinelli DR, Vita F, Adamo FC, Logrippo S, Parlapiano M, Bonacucina G, Pucciarelli S, Francescangeli O, Vaccari L, Pisani M (2021) Cubic and hexagonal mesophases for protein encapsulation: structural effects of insulin confinement. Langmuir 37(34):10166–10180. 10.1021/acs.langmuir.1c01587 PMC8397388 34369787

[CR3] Avila MC, Rade LL, Souza AS, Melo RR, Oliveira CC, Moraes MR, Silva EED, Generoso WC, Persinoti GF, Ramos CHI, Makris TM, Zanphorlin LM (2026) Temperature-dependent conformational dynamics govern regioselectivity in a CYP152 decarboxylase. J Biol Chem 302(4):111309. 10.1016/j.jbc.2026.111309 PMC13049924 41759725

[CR4] Barriga HMG, Holme MN, Stevens MM (2019) Cubosomes: the next generation of smart lipid nanoparticles? Angew Chem Int Ed 58(10):2958–2978. 10.1002/anie.201804067 PMC6606436 29926520

[CR5] Caffrey M (2015) A comprehensive review of the lipid cubic phase or in meso method for crystallizing membrane and soluble proteins and complexes. Acta Crystallogr Sect F. 10.1107/S2053230X14026843 PMC4304740 25615961

[CR6] Caffrey M, Cherezov V (2009) Crystallizing membrane proteins using lipidic mesophases. Nat Protoc 4(5):706–731. 10.1038/nprot.2009.31 PMC2732203 19390528

[CR7] Caselli L, Conti L, De Santis I, Berti D (2024) Small-angle X-ray and neutron scattering applied to lipid-based nanoparticles: recent advancements across different length scales. Adv Coll Interface Sci 327:103156. 10.1016/j.cis.2024.103156 38643519

[CR8] Catalini S, Rutsch M, Lapini A, Rossi B, Di Donato M, Bracco B, Paolantoni M, Yao Y (2026) Geometry controls confined water dynamics in lipidic mesophases. Angew Chem Int Ed 65. 10.1002/anie.202522757 PMC12887620 41518242

[CR9] Chen W-H, Vazquez-Gonzalez M, Zoabi A, Abu-Reziq R, Willner I (2018) Biocatalytic cascades driven by enzymes encapsulated in metal-organic framework nanoparticles. Nat Catal 1(9):689–695. 10.1038/s41929-018-0117-2

[CR10] Demurtas D, Guichard P, Martiel I, Mezzenga R, Hébert C, Sagalowicz L (2015) Direct visualization of dispersed lipid bicontinuous cubic phases by cryo-electron tomography. Nat Commun 6:8915. 10.1038/ncomms9915 PMC4660369 26573367

[CR11] Donida B, Tauffner B, Raabe M, Immich MF, Farias MA, Coutinho DDS, Machado AZ, Kessler RG, Portugal RV, Bernardi A, Frozza R, Moura DJ, Poletto F, Vargas CR (2018) Monoolein-based nanoparticles for drug delivery to the central nervous system: a platform for lysosomal storage disorder treatment. Eur J Pharm Biopharm 133:96–103. 10.1016/j.ejpb.2018.10.005 30315863

[CR12] Eczacioglu N, Asim MH, Haddadzadegan S, Bernkop-Schnürch A (2026) Liquid crystalline inverted lipid phases and reverse micelles in drug delivery: from molecular design to therapeutic potential. Adv Func Mater. 10.1002/adfm.202522873

[CR13] Farhan M, Hasani IW, Khafaga DSR, Ragab WM, Kazi RNA, Aatif M, Muteeb G, Fahim YA (2025) Enzymes as catalysts in industrial biocatalysis: advances in engineering, applications, and sustainable integration. Catalysts. 10.3390/catal15090891

[CR14] Fong WK, Salentinig S, Prestidge CA, Mezzenga R, Hawley A, Boyd BJ (2014) Generation of geometrically ordered lipid-based liquid-crystalline nanoparticles using biologically relevant enzymatic processing. Langmuir 30(19):5373–5377. 10.1021/la5003447 24783947

[CR15] Freire RVM, Tran B, Debas M, Zabara M, Amenitsch H, Salentinig S (2024) Nanostructure formation in glycerolipid films during enzymatic hydrolysis: a GISAXS study. ACS Appl Mater Interf 16(44):61262–61271. 10.1021/acsami.4c12125 39448890

[CR16] Generoso WC, Alvarenga AHS, Simões IT, Miyamoto RY, Melo RRd, Guilherme EPX, Mandelli F, Santos CA, Prata R, Santos CRd, Colombari FM, Morais MAB, Pimentel Fernandes R, Persinoti GF, Murakami MT, Zanphorlin LM (2025) Coordinated conformational changes in P450 decarboxylases enable hydrocarbons production from renewable feedstocks. Nat Commun 16(1):945. 10.1038/s41467-025-56256-4 PMC11754895 39843428

[CR17] Gilbert J, Valldeperas M, Dhayal SK, Barauskas J, Dicko C, Nylander T (2019) Immobilisation of -galactosidase within a lipid sponge phase: structure, stability and kinetics characterisation. Nanoscale 11(44):21291–21301. 10.1039/c9nr06675f 31667477

[CR18] Grippo V, Ma S, Ludwig R, Gorton L, Bilewicz R (2019) Cellobiose dehydrogenase hosted in lipidic cubic phase to improve catalytic activity and stability. Bioelectrochemistry 125:134–141. 10.1016/j.bioelechem.2017.10.003 29128298

[CR19] Holyavka MG, Artyukhov VG (2025) Prospects and problems in enzyme immobilization methodology: comprehensive review. Biophys Rev 17:1471. 10.1007/s12551-025-01360-4 PMC12847602 41613885

[CR20] Holyavka MG, Goncharova SS, Redko YA, Lavlinskaya MS, Sorokin AV, Artyukhov VG (2023) Novel biocatalysts based on enzymes in complexes with nano- and micromaterials. Biophys Rev 15(5):1127–1158. 10.1007/s12551-023-01146-6 PMC10643816 37975005

[CR21] Hong L, Salentinig S, Hawley A, Boyd BJ (2015) Understanding the mechanism of enzyme-induced formation of lyotropic liquid crystalline nanoparticles. Langmuir 31(24):6933–6941. 10.1021/acs.langmuir.5b01615 26029994

[CR22] Hyde S (2001) Identification of lyotropic liquid crystalline mesophases. In: Holmberg K (ed) Handbook of Applied Surface and Colloid Chemistry vol 1, 1st edn, pp 299–332. John Wiley & Sons, Chichester, England

[CR23] Israelachvili JN, Mitchell DJ, Ninham BW (1977) Theory of self-assembly of lipid bilayers and vesicles. Biochem Biophys Acta 470:185–201. 10.1016/0005-2736(77)90099-2 911827

[CR24] Kim H, Sung J, Chang Y, Alfeche A, Leal C (2018) Microfluidics synthesis of gene silencing cubosomes. ACS Nano. 10.1021/acsnano.8b03770 PMC6876307 30081623

[CR25] Leu JSL, Teoh JJX, Ling ALQ, Chong J, Loo YS, Azmi IDM, Zahid NI, Bose RJC, Madheswaran T (2023) Recent advances in the development of liquid crystalline nanoparticles as drug delivery systems. Pharmaceutics 15(5):1421. 10.3390/pharmaceutics15051421 PMC10224018 37242663

[CR26] Li D, Caffrey M (2011) Lipid cubic phase as a membrane mimetic for integral membrane protein enzymes. Proc Natl Acad Sci USA 108(21):8639–8644. 10.1073/pnas.1101815108 PMC3102357 21555546

[CR27] Li D, Caffrey M (2020) Structure and functional characterization of membrane integral proteins in the lipid cubic phase. J Mol Biol 432(18):5104–5123. 10.1016/j.jmb.2020.02.024 32113953

[CR28] Liu H, Wang Y, Wang Q, Li Z, Zhou Y, Zhang Y, Li S (2013) Protein-bearing cubosomes prepared by liquid precursor dilution: inner ear delivery and pharmacokinetic study following intratympanic administration. J Biomed Nanotechnol 9(10):1784–1793. 10.1166/jbn.2013.1685 24015508

[CR29] Liu W, Lewis SE, Lorenzo M, Squires AM (2024) Development of redox-active lyotropic lipid cubic phases for biosensing platforms. Langmuir 40(1):170–178. 10.1021/acs.langmuir.3c02307 PMC10786026 38113389

[CR30] Ma X, Pronay TS, Gao B, Zhao J (2025) Nanoengineered enzyme immobilization: toward biomedical, orthopedic, and biofuel applications. ACS Omega 10(32):35434–35450. 10.1021/acsomega.5c05589 PMC12368629 40852245

[CR31] Maghraby YR, El-Shabasy RM, Ibrahim AH, Azzazy HME-S (2023) Enzyme immobilization technologies and industrial applications. ACS Omega 8(6):5184–5196. 10.1021/acsomega.2c07560 PMC9933091 36816672

[CR32] Manni LS, Fong W-K, Mezzenga R (2020) Lipid-based mesophases as matrices for nanoscale reactions. Nanoscale Horizons 5(6):914–927. 10.1039/d0nh00079e 32322863

[CR33] Mariani P, Luzzati V, Delacroix H (1988) Cubic phases of lipid-containing systems: structure analysis and biological implications. J Mol Biol 204(1):165–189. 10.1016/0022-2836(88)90607-9 3216391

[CR34] Mazzoni S, Barbosa LRS, Funari SS, Itri R, Mariani P (2016) Cytochrome-c affects the monoolein polymorphism: consequences for stability and loading efficiency of drug delivery systems. Langmuir. 10.1021/acs.langmuir.5b03507 26710233

[CR35] Mendozza M, Balestri A, Montis C, Berti D (2020) Controlling the kinetics of an enzymatic reaction through enzyme or substrate confinement into lipid mesophases with tunable structural parameters. Int J Mol Sci 21(14):5116. 10.3390/ijms21145116 PMC7404178 32698376

[CR36] Mertins O, Mathews PD, Angelova A (2020) Advances in the design of ph-sensitive cubosome liquid crystalline nanocarriers for drug delivery applications. Nanomaterials 10(5):963. 10.3390/nano10050963 PMC7281514 32443582

[CR37] Mezzenga R (2023) Challenges and opportunities from water under soft nanoconfinement. Front Soft Matter. 10.3389/frsfm.2023.1324589

[CR38] Mezzenga R, Seddon JM, Drummond CJ, Boyd BJ, Schröder-Turk GE, Sagalowicz L (2019) Nature-inspired design and application of lipidic lyotropic liquid crystals. Adv Mater 31:1900818. 10.1002/adma.201900818 31222858

[CR39] Mishraki T, Libster D, Aserin A, Garti N (2010) Temperature-dependent behavior of lysozyme within the reverse hexagonal mesophases (H). Colloids Surf B 75(2):391–397. 10.1016/j.colsurfb.2009.08.052 19836212

[CR40] Miyamoto RY, Melo RR, Mesquita Sampaio IL, Sousa AS, Morais ER, Sargo CR, Zanphorlin LM (2022) Paradigm shift in xylose isomerase usage: a novel scenario with distinct applications. Crit Rev Biotechnol 42(5):693–712. 10.1080/07388551.2021.1962241 34641740

[CR41] Mondal S, Bagchi B (2024) Dielectric properties of nanoconfined water. J Chem Phys 161:220901. 10.1063/5.0234293 39655679

[CR42] Nagarajan R (2002) Molecular packing parameter and surfactant self-assembly: the neglected role of the surfactant tail. Langmuir. 10.1021/la010831y

[CR43] Nazaruk E, Bilewicz R (2007) Catalytic activity of oxidases hosted in lipidic cubic phases on electrodes. Bioelectrochemistry 71(1):8–14. 10.1016/j.bioelechem.2006.12.007 17289444

[CR44] Nazaruk E, Bilewicz R, Lindblom G, Lindholm-Sethson B (2008) Cubic phases in biosensing systems. Anal Bioanal Chem 391:1569–1578. 10.1007/s00216-008-2149-y 18488208

[CR45] Nazaruk E, Landau EM, Bilewicz R (2014) Membrane bound enzyme hosted in liquid crystalline cubic phase for sensing and fuel cells. Electrochim Acta 140:96–100. 10.1016/j.electacta.2014.05.130

[CR46] Palma AS, Casadei BR, Lotierzo MC, Castro RD, Barbosa LRS (2023) A short review on the applicability and use of cubosomes as nanocarriers. Biophys Rev 15(4):553–567. 10.1007/s12551-023-01089-y PMC10480096 37681099

[CR47] Peters RJRW, Marguet M, Marais S, Fraaije MW, Hest JCM, Lecommandoux S (2014) Cascade reactions in multicompartmentalized polymersomes. Angew Chem Int Ed 53(1):146–150. 10.1002/anie.201308141 24254810

[CR48] Pilkington CP, Contini C, Barritt JD, Simpson PA, Seddon JM, Elani Y (2023) A microfluidic platform for the controlled synthesis of architecturally complex liquid crystalline nanoparticles. Sci Rep. 10.1038/s41598-023-39205-3 PMC10403506 37542147

[CR49] Porta AP, Rodriguez-Torres MI, Hilbert CR, Woodley JM, Erdem E (2026) Interface-aware design of biocatalysis. Adv Synth Catal. 10.1002/adsc.70392

[CR50] Portmann M, Landau EM, Luisi PL (1991) Spectroscopic and rheological studies of enzymes in rigid lipidic matrices: the case of alpha-chymotrypsin in a lysolecithin-water cubic phase. J Phys Chem 95(22):8437–8440. 10.1021/j100175a006

[CR51] Qiu H, Caffrey M (2000) The phase diagram of the monoolein/water system: metastability and equilibrium aspects. Biomaterials 21(3):223–234. 10.1016/S0142-9612(99)00126-X 10646938

[CR52] Rade LL, Generoso WC, Das S, Souza AS, Silveira RL, Avila MC, Vieira PS, Miyamoto RY, Lima ABB, Aricetti JA, Melo RR, Milan N, Persinoti GF, Bonomi AMFLJ, Murakami MT, Makris TM, Zanphorlin LM (2023) Dimer-assisted mechanism of (un)saturated fatty acid decarboxylation for alkene production. Proceed Nat Acad Sci 120(22):2221483120. 10.1073/pnas.2221483120 PMC10235961 37216508

[CR53] Rakotoarisoa M, Angelov B, Espinoza S, Khakurel K, Bizien T, Angelova A (2019) Cubic liquid crystalline nanostructures involving catalase and curcumin: BioSAXS study and catalase peroxidatic function after cubosomal nanoparticle treatment of differentiated SH-SY5Y cells. Molecules 24(17):3058. 10.3390/molecules24173058 PMC6749324 31443533

[CR54] Ramberg KO, Boland C, Kooshapur H, Soubias O, Wiktor M, Huang C-Y, Bailey J, Gawrisch K, Caffrey M (2025) Solid-state NMR of membrane peptides and proteins in the lipid cubic phase. Biophys J 124(9):1387–1400. 10.1016/j.bpj.2025.03.012 PMC12256886 40119522

[CR55] Raw J, Franco LR, Rodrigues LFdC, Barbosa LRS (2023) Unveiling the three-step model for the interaction of imidazolium-based ionic liquids on albumin. ACS Omega 8:38101. 10.1021/acsomega.3c04188 PMC10586182 37867681

[CR56] Razumas V, Kanapieniene J, Nylander T, Engstrom S, Larsson K (1994) Electrochemical biosensors for glucose, lactate, urea, and creatinine based on enzymes entrapped in a cubic liquid-crystalline phase. Anal Chim Acta 289(2):155–162. 10.1016/0003-2670(94)80098-7

[CR57] Razumas V, Talaikyte Z, Barauskas J, Miezis Y, Nylander T (1997) An FT-IR study of the effects of distearoylphosphatidylglycerol and cytochrome c on the molecular organization of the monoolein-water cubic liquid-crystalline phase. Vib Spectrosc 15(1):91–101. 10.1016/S0924-2031(97)00021-0

[CR58] Ribeiro IRS, Harkiolaki M, Plivelic TS, Barbosa LRS (2025) Super-resolution fluorescence microscopy and cryogenic soft X-ray tomography enable intracellular localization of stable nanoparticles. Nano Today 61:102599. 10.1016/j.nantod.2024.102599

[CR59] Sarkar S, Tran N, Rashid MH, Le TC, Yarovsky I, Conn CE, Drummond CJ (2019) Toward cell membrane biomimetic lipidic cubic phases: a high-throughput exploration of lipid compositional space. ACS Appl Bio Mater 2(1):182–195. 10.1021/acsabm.8b00539 35016341

[CR60] Sheldon RA, Pelt S (2013) Enzyme immobilisation in biocatalysis: why, what and how. Chem Soc Rev. 10.1039/C3CS60075K 23532151

[CR61] Spinozzi F, Ferrero C, Ortore MG, De Maria Antolinos A, Mariani P (2014) GENFIT: software for the analysis of small-angle X-ray and neutron scattering data of macro-molecules in solution. J Appl Crystallogr 47(3):1132–1139. 10.1107/S1600576714005147 PMC4038801 24904247

[CR62] Strachan JB, Dyett BP, Jones NC, Hoffmann SV, Valery C, Conn CE (2021) Reduction of enzymatic degradation of insulin via encapsulation in a lipidic bicontinuous cubic phase. J Colloid Interface Sci 592:135–144. 10.1016/j.jcis.2021.02.027 33647562

[CR63] Sulis DB, Lavoine N, Sederoff H, Jiang X, Marques BM, Lan K, Cofre-Vega C, Barrangou R, Wang JP (2025) Advances in lignocellulosic feedstocks for bioenergy and bioproducts. Nat Commun. 10.1038/s41467-025-56472-y PMC11787297 39893176

[CR64] Sun W, Vallooran JJ, Zabara A, Mezzenga R (2014) Controlling enzymatic activity and kinetics in swollen mesophases by physical nano-confinement. Nanoscale 6(12):6853–6859. 10.1039/c4nr01394h 24831024

[CR65] Sun W, Vallooran JJ, Mezzenga R (2015) Enzyme kinetics in liquid crystalline mesophases: size matters. But Also Topology Langmuir 31(15):4558–4565. 10.1021/acs.langmuir.5b00579 25806598

[CR66] Sun W, Vallooran JJ, Fong W-K, Mezzenga R (2016) Lyotropic liquid crystalline cubic phases as versatile host matrices for membrane-bound enzymes. J Phys Chem Lett 7(8):1507–1512. 10.1021/acs.jpclett.6b00416 27050734

[CR67] Thorn CR, Raju D, Lacdao I, Gilbert S, Sivarajah P, Howell PL, Prestidge CA, Thomas N (2021) Protective liquid crystal nanoparticles for targeted delivery of PslG: a biofilm dispersing enzyme. ACS Infect Diseas 7(8):2102–2115. 10.1021/acsinfecdis.1c00014 33908759

[CR68] Valldeperas M, Salis A, Barauskas J, Tiberg F, Arnebrant T, Razumas V, Monduzzi M, Nylander T (2019a) Enzyme encapsulation in nanostructured self-assembled structures: toward biofunctional supramolecular assemblies. Current Opin Coll Interf Sci 44:130–142. 10.1016/j.cocis.2019.09.007

[CR69] Valldeperas M, Wisniewska M, Ram-On M, Kesselman E, Danino D, Nylander T, Barauskas J (2016) Sponge phases and nanoparticle dispersions in aqueous mixtures of mono- and diglycerides. Langmuir 32(34):8650–8659. 10.1021/acs.langmuir.6b01356 27482838

[CR70] Valldeperas M, Talaikis M, Dhayal SK, Velicka M, Barauskas J, Niaura G, Nylander T (2019b) Encapsulation of aspartic protease in nonlamellar lipid liquid crystalline phases. Biophys J 117(5):829–843. 10.1016/j.bpj.2019.07.031 PMC6732526 31422820

[CR71] Valli M, Russo HM, Bolzani VS (2018) The potential contribution of the natural products from Brazilian biodiversity to bioeconomy. An Acad Bras Ciênc. 10.1590/0001-3765201820170653 29668803

[CR72] Vallooran JJ, Handschin S, Pillai SM, Vetter BN, Rusch S, Beck H-P, Mezzenga R (2016) Lipidic cubic phases as a versatile platform for the rapid detection of biomarkers, viruses, bacteria, and parasites. Adv Func Mater 26(2):181–190. 10.1002/adfm.201503428

[CR73] van’t Hag L, Anandan A, Seabrook SA, Gras SL, Drummond CJ, Vrielink A, Conn CE (2017) Direct demonstration of lipid phosphorylation in the lipid bilayer of the biomimetic bicontinuous cubic phase using the confined enzyme lipid A phosphoethanolamine transferase. Soft Matter 13(7):1493–1504. 10.1039/c6sm02487d 28125111

[CR74] Vivian JT, Callis PR (2001) Mechanisms of tryptophan fluorescence shifts in proteins. Biophys J 80:2093–2109. 10.1016/S0006-3495(01)76183-8 PMC1301402 11325713

[CR75] Vriezema DM, Garcia PML, Oltra NS, Hatzakis NS, Kuiper SM, Nolte RJM, Rowan AE, Hest JCM (2007) Positional assembly of enzymes in polymersome nanoreactors for cascade reactions. Angew Chem Int Ed 46(39):7378–7382. 10.1002/anie.200701125 17705203

[CR76] Wohlgemuth R (2024) Enzyme catalysis for sustainable value creation using renewable biobased resources. Molecules 29(23):5772. 10.3390/molecules29235772 PMC11643197 39683928

[CR77] Yao Y, Zhou T, Farber R, Grossner U, Floudas G, Mezzenga R (2021) Designing cryo-enzymatic reactions in subzero liquid water by lipidic mesophase nanoconfinement. Nat Nanotechnol 16(7):802–810. 10.1038/s41565-021-00893-5 33941918

[CR78] Zabara A, Chong JTY, Martiel I, Stark L, Cromer BA, Speziale C, Drummond CJ, Mezzenga R (2018) Design of ultra-swollen lipidic mesophases for the crystallization of membrane proteins with large extracellular domains. Nat Commun. 10.1038/s41467-018-02996-5 PMC5803273 29416037

[CR79] Zaborowska-Mazurkiewicz M, Nazaruk E, Bilewicz R (2025) Lipid liquid-crystalline nanoparticles as a suitable platform for accommodating sensitive membrane proteins: monitoring the activity of HMG-CoA reductase. J Nanobiotechnol 23(1):334. 10.1186/s12951-025-03370-6 PMC12057187 40336087

[CR80] Zatloukalova M, Nazaruk E, Novak D, Vacek J, Bilewicz R (2018) Lipidic liquid crystalline cubic phases for preparation of ATP-hydrolysing enzyme electrodes. Biosens Bioelectron 100:437–444. 10.1016/j.bios.2017.09.036 28961546

[CR81] Zatloukalova M, Nazaruk E, Bilewicz R (2019) Electrogenic transport of Na/K-ATPase incorporated in lipidic cubic phases as a model biomimetic membrane. Electrochim Acta 310:113–121. 10.1016/j.electacta.2019.04.082

[CR82] Zhou T, Vallooran JJ, Assenza S, Szekrenyi A, Clapes P, Mezzenga R (2018) Efficient asymmetric synthesis of carbohydrates by aldolase nano confined in lipidic cubic mesophases. ACS Catal 8(7):5810–5815. 10.1021/acscatal.8b01716

[CR83] Zunzunegui-Bru E, Alfarano SR, Züblin P, Baraldi L, Vondracek H, Piccirilli F, Vaccari L, Mezzenga R (2025) Ultrafast intermolecular dynamics of nanoconfined water in swollen lipid cubic mesophases. Small 21. 10.1002/smll.202508744 PMC12632434 41043057

